# Preoperative oral carbohydrate administration in adult elective surgery: protocol heterogeneity, regional practice patterns and evidence gaps–a scoping review

**DOI:** 10.3389/fnut.2026.1943627

**Published:** 2026-09-11

**Authors:** Bang Xiao, Yuanfei Liu, Renqiang Lu, Ruyi Tan, Xianzhang Zeng, Xiuying Lu, Chang Yang

**Affiliations:** 1 Department of Anesthesiology, Chongqing University Cancer Hospital, Chongqing, China; 2 Sichuan Provincial Cancer Hospital, Chengdu, China

**Keywords:** enhanced recovery after surgery, evidence-based nursing, perioperative care, preoperative oral carbohydrate, scoping review

## Abstract

**Objective:**

This scoping review aimed to map the extent, characteristics and distribution of evidence on preoperative oral carbohydrate administration in adult elective surgical patients. Specifically, we sought to describe publication trends, regional practice patterns, surgical populations, intervention protocols, outcome domains and evidence gaps, thereby informing future standardization and individualized implementation within ERAS pathways.

**Methods:**

Following the Arksey and O’Malley framework for scoping reviews, we searched eight Chinese and English databases including CNKI, Wanfang, PubMed, and Web of Science, to collect original studies published from 2015 to September 2025 on preoperative oral carbohydrate intake. Studies were screened and data extracted according to predetermined inclusion and exclusion criteria, followed by descriptive analysis.

**Results:**

Eighty-two studies were included, comprising 76 randomized controlled trials and 6 non-randomized studies. Most studies were conducted in China and other Asian settings, and more than two-thirds were published after 2020. The commonly reported regimen involved 200–400 mL of a carbohydrate-containing clear fluid administered 2–3 h before surgery, although formulation, dose, timing and control conditions varied substantially across studies. Outcomes were mapped into five domains: metabolic responses, patient comfort, inflammatory or stress markers, clinical recovery and safety. Most included studies reported favorable metabolic or comfort-related findings; however, evidence was concentrated in single-center trials, short-term outcomes and relatively low-risk elective surgical populations.

**Conclusion:**

Current literature suggests that preoperative oral carbohydrate administration is a widely studied and feasible component of ERAS pathways, but the evidence base remains heterogeneous in intervention protocols, outcome definitions and population coverage. Future multicenter studies should standardize reporting, include higher-risk populations such as older adults, frail patients and patients with diabetes, and assess long-term functional and patient-centered outcomes.

**Systematic review registration:**

https://osf.io/9rd68/

## Introduction

Surgery constitutes a core therapeutic strategy in modern medicine and is widely applied clinically. Data from the 2018 US National Health Interview Survey (NHIS) reported an annual surgical rate of approximately 11.3% (95% CI: 10.8%–11.7%) in the general population, rising to 19.4% among adults aged ≥65 years and 12.3% in women. Surgery-related medical expenditure accounts for 26.3% of total healthcare spending in the United States, reflecting its vital position and massive resource occupation in healthcare systems (1). Although surgery is an effective treatment modality, its inherent invasiveness can trigger multiple pathophysiological responses in the body. Intraoperative tissue injury, ischemia-reperfusion, and anesthetic interventions collectively activate oxidative stress pathways, leading to excessive accumulation of reactive oxygen species (ROS). This subsequently depletes antioxidant defense systems–manifested as significantly reduced total antioxidant capacity (T-AOC) and reduced glutathione (GSH) levels–while increasing oxidative damage markers such as malondialdehyde (MDA) and 8-hydroxy-2′-deoxyguanosine (8-OHdG). This oxidative imbalance has been shown to be closely associated with increased postoperative infection risk, delayed wound healing, perioperative cardiovascular events, and elevated long-term mortality (2). Moreover, surgical stress rapidly induces peripheral insulin resistance and acute postoperative hyperglycemia. This metabolic disorder amplifies inflammation and oxidative stress, inhibits tissue repair and weakens immune function. Multiple cohort studies have confirmed its association with prolonged hospital stay, delayed wound recovery and increased postoperative infection risk (3). To alleviate surgical trauma-related pathophysiological disorders and optimize perioperative care, the Enhanced Recovery After Surgery (ERAS) protocol has been developed and globally popularized. First proposed systematically in 1997 by Danish surgeon Prof. Henrik Kehlet, ERAS adopts a multidisciplinary model integrating evidence-based perioperative measures to minimize physical and psychological stress responses. Its core goals include shortening length of hospital stay, reducing medical resource consumption and lowering postoperative complication rates (4). After decades of development, ERAS has been widely adopted worldwide. In 2018, China released the “Chinese Expert Consensus and Pathway Management Guidelines for Enhanced Recovery After Surgery,” further promoting standardized clinical implementation of this concept within the country (5).

Preoperative oral carbohydrate (POC) loading is an essential ERAS measure for elective surgical patients, referring to preoperative intake of isotonic clear solutions containing glucose, maltodextrin and other components. The formulation maintains isotonicity to avoid gastrointestinal discomfort caused by osmotic disturbance, and standardized administration timing supports energy supply without delaying gastric emptying (6, 7). As a key component of the enhanced recovery concept, preoperative oral carbohydrate intake has fundamentally shifted the traditional practice of prolonged preoperative fasting and nil-by-mouth regimens–historically requiring patients to fast for 8 h and abstain from clear fluids for 6 h prior to surgery, which often induces hunger, thirst, anxiety, and exacerbates postoperative insulin resistance (8). In contrast, preoperative oral carbohydrate administration–through timely and physiologically appropriate energy supplementation–effectively mitigates these issues: it not only alleviates preoperative hunger, thirst, and fatigue but also enhances patient comfort (9). Moreover, it effectively reduces the incidence of postoperative insulin resistance, attenuates oxidative stress, lowers the risk of postoperative infection, and shortens hospital stay (3). And it does not delay gastric emptying or increase the risk of intraoperative aspiration, thereby ensuring surgical safety (9).

The clinical value of this intervention has been endorsed by authoritative guidelines worldwide: the American Society of Anesthesiologists (ASA) explicitly recommends, in its 2023 updated preoperative fasting guidelines, that healthy elective surgery patients may consume carbohydrate-containing clear liquids up to 2 h before surgery–citing robust evidence demonstrating reduced preoperative hunger without increased risk of adverse outcomes (8). Similarly, the European Society for Clinical Nutrition and Metabolism (ESPEN) 2025 Clinical Nutrition Guidelines for Surgery recommend oral administration of 800 mL of a 12.5% carbohydrate solution 10 h before surgery and an additional 400 mL 2 h before surgery (6). The Chinese Guidelines for Clinical Application of Parenteral and Enteral Nutrition in Adult Patients (2023 Edition) similarly emphasize that preoperative carbohydrate loading mitigates surgical stress and improves insulin resistance, and explicitly advise against overnight fasting protocols (7).

Although the clinical benefits of preoperative oral carbohydrate administration have been extensively validated, current evidence remains heterogeneous across research focus, intervention protocols, and clinical implementation: Critical intervention variables–including applicable surgical procedures, optimal timing of intake, solution concentration, and administered dose–exhibit substantial heterogeneity across studies, and clinical practice varies significantly across geographic regions; consequently, the existing evidence base remains fragmented and incompletely synthesized (3). Therefore, this scoping review aims to systematically map the English- and Chinese-language evidence published over the past decade, clarify the impact of each critical intervention variable on clinical outcomes, identify existing evidence gaps, and thereby inform the standardization and personalization of preoperative oral carbohydrate administration–while also providing a clear conceptual and methodological framework for future targeted research.

## Methods

### Research questions and protocol registration

This scoping review adheres to the methodological framework for scoping studies proposed by Arksey and O’Malley (10). This framework, first introduced in 2005, explicitly defines four core purposes of scoping studies: (i) to map the breadth, extent, and nature of existing research activity; (ii) to determine the value of conducting a full systematic review; (iii) to summarize and disseminate research findings; and (iv) to identify evidence gaps in the existing literature (10). As a seminal methodological paradigm in the field of scoping studies, this framework has subsequently undergone practical refinements by multiple scholars: for instance, Tricco et al. integrated it with reporting standards in their 2018 PRISMA Extension for Scoping Studies (PRISMA-ScR), thereby enhancing the transparency and reproducibility of scoping reviews (11); Munn et al. further refined the operational procedures of this framework in their 2018 study, emphasizing that clearly formulated research questions serve to anchor the core dimensions of the scoping review and thereby enhance its conceptual coherence (12). This study adheres to the core methodological logic of the Arksey framework while incorporating recent refinements–particularly those emphasizing conceptual clarity and operational rigor–to specifically explore the application of preoperative oral carbohydrate administration within perioperative nursing practice.

1. What are the defining characteristics of current research activity on preoperative oral carbohydrate administration in the perioperative setting? Specifically, what are the geographic distribution patterns, the spectrum of surgical procedures addressed, the predominant study designs (e.g., proportions of single-center vs. multicenter trials, use of blinding), and the distribution of sample sizes across studies?
2. What are the key sources of heterogeneity in preoperative oral carbohydrate intervention protocols? Specifically, how do clinical practices vary with respect to carbohydrate type, solution concentration, administered dose, and timing of intake–and how do these protocol elements align with specific surgical procedures and patient populations (e.g., individuals with vs. without diabetes)?
3. What is the current state of evidence regarding the effects of preoperative oral carbohydrate administration on core perioperative outcomes? Specifically, what do studies report concerning its impact on metabolic parameters (e.g., blood glucose levels, insulin resistance), inflammatory biomarkers, patient-reported perioperative comfort, and clinical endpoints (e.g., postoperative complication rates, length of hospital stay)–and to what extent do findings demonstrate consistency or divergence across studies?
4. What evidence gaps persist in the literature on preoperative oral carbohydrate administration? Specifically, which patient subgroups remain underrepresented (e.g., older adults, frail individuals, or those with complex comorbidities), which critical outcomes are consistently omitted (e.g., long-term functional recovery, health-related quality of life, or postoperative cognitive dysfunction), and which intervention protocol variants lack systematic comparative evaluation (e.g., different carbohydrate formulations, dosing regimens, or timing strategies)?

To ensure transparency, methodological rigor, and reproducibility, this scoping review has been prospectively registered on the Open Science Framework (OSF) under registration DOI: 10.17605/OSF.IO/9RD68.

### Eligibility criteria

To establish a consistent boundary for the core concept of this study, we first define and clarify “preoperative oral carbohydrate loading”: in this study, preoperative oral carbohydrate loading refer to carbohydrate-containing preparations (including maltodextrin, glucose, commercial specialized carbohydrate drinks, etc.) administered orally to patients during the perioperative period before surgery begins. These are taken either the day before surgery or within 2–3 h prior to surgery on the day of surgery (either as a single dose or multiple doses), with the preparation’s carbohydrate concentration and dosage clearly specified (13). This definition encompasses the mainstream intervention approaches currently used in clinical practice and aligns with the recommended scope outlined in Enhanced Recovery After Surgery (ERAS) guidelines (5). Based on the above core concept definitions, this study systematically established and clarified the following inclusion and exclusion criteria.

### Inclusion criteria

➀ Aged 18 years or older, non-pregnant, and non-lactating; ➁ Scheduled to undergo elective surgery, with no definitive contraindications to preoperative oral carbohydrate administration, and clinically evaluated to have swallowing and digestive functions compatible with oral intake; ➂ Intervention: Preoperative oral carbohydrate administration, with dosing administered on the day prior to surgery and 2–3 h preoperatively on the day of surgery, via the oral route. ➃ Study design: Primary studies, including randomized controlled trials (RCTs), prospective cohort studies, retrospective cohort studies, and case-control studies; ➄ Outcome measures: Studies must report at least one outcome measure. Preoperative outcomes include hunger scores and thirst scores; postoperative outcomes include insulin resistance, incidence of nausea and vomiting, time to first flatus or bowel movement, length of hospital stay, and infection rate. ➅ Language and availability: The literature is in Chinese or English, with full-text access available.

### Exclusion criteria

➀ Presence of end-stage organ failure, including Child-Pugh class C cirrhosis, stage 5 chronic kidney disease without dialysis, and acute respiratory failure requiring mechanical ventilation. ➁ Non-compliant intervention regimens: Intravenous administration of nutritional supplements or glucose preoperatively, combined intravenous infusion of glucose or nutritional supplements during the intervention period, or preoperative provision of non-clear beverage. ➂ Preoperative confounding factors: Administration of oral antibiotics between 1 day and 2–3 h preoperatively; Use of corticosteroids or non-basal insulin (for non-basal glycemic control) within 1 week prior to surgery; Strict carbohydrate restriction (e.g., low-carbohydrate diet) within 1 week preoperatively. ➃ Surgery-related factors: Severe intraoperative complications (e.g., massive bleeding, cardiac arrest) leading to incomplete postoperative recovery data. ➄ Study quality non-compliance: sample size <20 cases, outcome indicator data missing rate >30% without reasonable handling methods (e.g., multiple imputation), lack of explicit reporting on oral carbohydrate dose, concentration, and administration time, duplicate publications (only the most recent or highest-quality article retained), and studies with explicit but undisclosed conflicts of interest. The minimum sample size threshold of 20 participants was adopted to exclude severely underpowered single-case reports and small pilot studies, which tend to provide unstable effect estimates and lack comprehensive descriptions of intervention implementation. Additionally, studies exclusively recruiting patients with decompensated diabetes, morbid obesity or advanced liver disease were excluded due to inconsistent safety monitoring protocols in preliminary investigations. Studies without disclosed conflicts of interest were also excluded to lower the potential risk of biased outcome reporting. We acknowledge that these predefined restrictions inevitably narrow the breadth of evidence mapping.

Inclusion and exclusion criteria were independently evaluated by two researchers, with discrepancies resolved via third-party arbitration. For items involving “clinical assessment” or “diagnostic criteria” (e.g., gastric emptying dysfunction, swallowing dysfunction), the diagnostic basis or assessment tools explicitly reported in the original studies were taken as the reference; studies failing to report such information were excluded.

### Search strategy

Based on the aforementioned conceptual definitions and pre-specified inclusion and exclusion criteria, the search strategy was designed to balance comprehensiveness and precision. Chinese databases–including CNKI, Wanfang Data, VIP, and SinoMed–were searched, alongside English-language databases–PubMed, Web of Science, Embase, and the Cochrane Library. The search covered all records from database inception to 30 September 2025. Synonyms and related terms were systematically expanded using controlled vocabulary (e.g., MeSH, Emtree) and free-text terms to maximize sensitivity without compromising specificity. The Chinese search strategy was constructed as follows: Preoperative AND (oral + drinking) AND (carbohydrate + carbohydrate nutrition + energy drink + carbohydrate drink) NOT (Review + systematic review + meta + case report + expert consensus + commentary + Animal experiment) (in Chinese). The English search strategy was: (Preoperative OR Pre-operation OR “Pre-op”) AND (Oral OR Ingestion OR Drinking OR “Oral intake”) AND (Carbohydrate* OR CHO OR “Carbohydrate supplement*” OR “Carbohydrate nutritional supplement*” OR “Functional beverage*” OR “Functional drink*” OR “Carbohydrate beverage*” OR “Carbohydrate drink*” OR “Carbohydrate fluid*” OR “Oral carbohydrate loading” OR OCL) NOT (SR OR “Systematic review” OR MA OR “Meta-analysis” OR Review* OR “Review article” OR “Case report” OR “Case study” OR “Expert consensus” OR “Expert agreement” OR Commentary OR Editorial OR “Animal experiment*” OR “Animal study*” OR “Animal research*”).

The literature search was independently performed by two researchers trained in evidence-based nursing methodology. A standardized operating protocol was uniformly implemented prior to the search; discrepancies regarding database-specific syntax were resolved through joint consultation to adjust the search strings. Search results were imported into EndNote X9 for automated deduplication, followed by manual cross-verification. The screening process adhered to a two-step workflow: initial screening of titles and abstracts → full-text re-screening. Discrepancies were reviewed and adjudicated by a third senior evidence-based nursing expert, further reinforcing the objectivity of the search and screening procedures.

## Results

### Literature search and screening results

**FIGURE 1:**
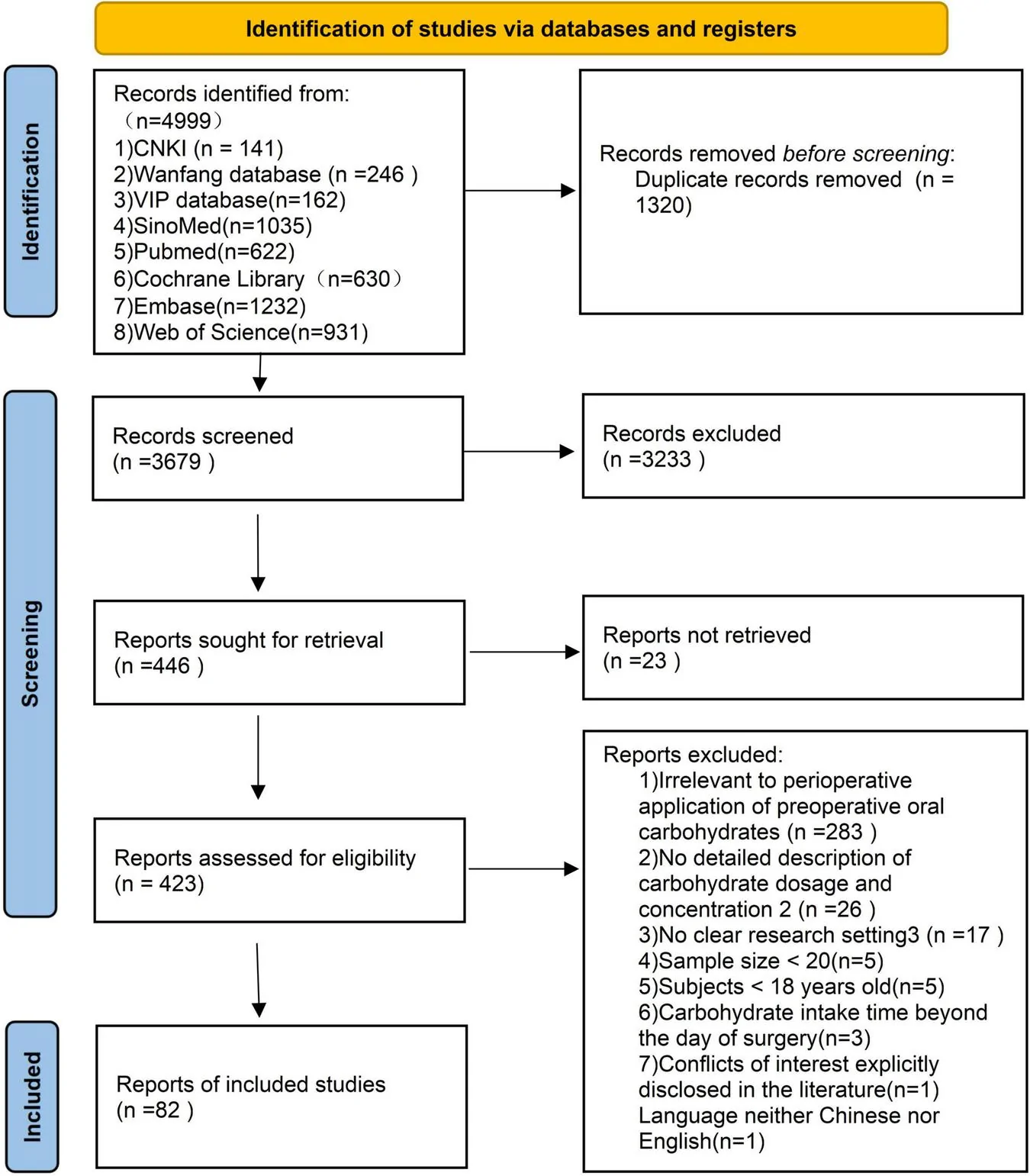
PRISMA 2020 flow diagram of the study selection process. Of 4,999 records identified from eight databases, 1,320 duplicates were removed; of 3,679 screened records, 3,233 were excluded; of 446 reports sought for retrieval, 23 were not retrieved; of 423 reports assessed for eligibility, 341 were excluded for reasons including irrelevance, insufficient detail, incorrect sample size and inappropriate subject age; 82 studies were ultimately included in the scoping review.

A comprehensive search across all target databases was conducted in strict adherence to the predefined search strategy, and the literature screening workflow followed PRISMA-ScR guidelines (Figure 1). Initial searches retrieved 4,999 records. After deduplication, 3,679 unique records remained. Title/abstract screening excluded 3,233 ineligible articles, leaving 446 manuscripts for full-text evaluation. After full-text assessment, 364 articles were excluded, and 82 publications were ultimately included for data charting. Primary full-text exclusion reasons were: irrelevant research topic (*n* = 283), incomplete reporting of carbohydrate dosage or concentration (*n* = 26), unavailable full text (*n* = 23), unclear study location (*n* = 17), sample size <20 (*n* = 5), participants younger than 18 years (*n* = 5), incorrect timing of carbohydrate intake (*n* = 3), undisclosed conflicts of interest (*n* = 1), non-Chinese/non-English language (*n* = 1). All extracted data were cross-checked against the original full texts by two reviewers. Discrepancies in study classification, intervention formulation and outcome categorization were resolved through consensus discussion with a third reviewer before final descriptive synthesis.

### Basic information of included studies

Data were charted using a standardized extraction form, including author, year, country or region, study design, setting, sample size, surgical specialty, patient characteristics, carbohydrate formulation, concentration, dose, timing, control condition, outcome domains and key findings (Table 1). The carbohydrate products used in the intervention groups of the included studies, together with their manufacturers and ingredient compositions, are summarized in Table 2. Two reviewers independently extracted data, and disagreements were resolved through discussion with a third reviewer. Findings were synthesized descriptively using frequencies and percentages for categorical variables and narrative summaries for intervention protocols, outcome domains and evidence gaps.

**TABLE 1:** Data extraction table.

No.	Title	References	Country/ region	Type of surgery	Sample size	Intervention measures of experimental group	Control group interventions	Outcome measures	Whether it is multicenter
1	The effect of preoperative oral carbohydrate on insulin resistance after hip replacement in elderly patients	Wang and Wang (22)	China	Hip replacement surgery	120 cases (carbohydrate group: 40; placebo group: 40; no-drinking group: 40)	Drink 500 mL of 10% glucose solution orally 2–3 h before anesthesia and finish it within 30 min.	Placebo group: drink an equal volume of distilled water 2–3 h before anesthesia. Fasting and no-drinking group: fast for 12 h and abstain from drinking for 8 h before surgery.	1. Subjective comfort: thirst, hunger, anxiety, nausea (VAS score, 0–100 points); 2. Gastric residual volume (nasogastric tube negative pressure suction measurement before anesthesia); 3. Blood glucose (peripheral finger blood test), insulin level (electrochemical method); 4. Insulin resistance index (HOMA-IR) and insulin sensitivity index (HOMA-ISI) (calculated by homeostasis model assessment); 5. Incision healing (grade A/B/C healing classification	N
2	Application of enhanced recovery after surgery (ERAS) in perioperative diet for elderly patients undergoing hip and knee replacement	Yu et al. (29)	China	Unilateral hip or knee replacement	84 cases (intervention group: 42; control group: 42)	1. The first operation: drink 200 mL of 25% maltodextrin solution orally 2 h before surgery. 2. The second operation: drink 200 mL of maltodextrin solution orally 2 h and 3–4 h before surgery. 3. The third and subsequent operations: drink 250 mL of residue-free predigested nutrient solution orally 6 h before surgery, and drink 200 mL of 25% maltodextrin solution orally 2 h before surgery.	Fast from food and drink after midnight on the night before surgery.	1. Safety indicators: incidence of intraoperative aspiration; 2. Gastrointestinal symptoms: incidence of postoperative nausea, vomiting, thirst, hunger and abdominal distension; 3. Rehabilitation indicators: postoperative anal exhaust time and hospital stay	N
3	Effect of preoperative oral carbohydrate solution on postoperative insulin resistance in patients undergoing laparoscopic surgery for rectal cancer	Yi et al. (19)	China	Laparoscopic radical resection of rectal cancer	70 cases (intervention group: 35; control group: 35)	Drink 400 mL of mixed carbohydrate solution orally 3 h before surgery.	Fast for 12 h and withhold water for 8 h before surgery.	1. Basic surgical information: operation time, intraoperative blood loss, intraoperative fluid infusion, and hospitalization time; 2. Blood glucose and insulin related indexes: fasting plasma glucose (FPG), fasting insulin, insulin resistance index, insulin sensitivity index, insulin growth factor (IGF-1) (1 day before operation and 1 day after operation); 3. The gray value of gluconeogenesis-related enzymes: the gray value of glucose-6-phosphate dehydrogenase (G-6-Pase) and phosphoenolpyruvate carboxykinase (PEPCK) in liver and small intestine (detected by Western blot); 4. Postoperative complications: abdominal infection, incision infection, pulmonary infection, anastomotic leakage, diarrhea, nausea and vomiting (recorded during hospitalization)	Y
4	A randomized controlled study of preoperative oral carbohydrate loading versus fasting in patients undergoing elective craniotomy	Liu et al. (33)	China	Craniotomy (involving meningioma, acoustic neuroma, glioma and other intracranial lesions resection)	120 cases (intervention group: 58; control group: 62)	Drink 400 mL of the Su Qian 2 h before the operation.	Preoperative fasting for 8 h	1. Primary outcome: glucose homeostasis (fasting blood glucose and insulin levels 4 h before surgery, upon entering the operating room, and 1–3 days after surgery); 2. Secondary outcomes: ➀ grip strength (before surgery and 1–3 days after surgery); ➁ pulmonary function (peak expiratory flow rate PEFR, before surgery and 1–3 days after surgery); ➂ postoperative complications (surgery-related: surgical site infection, intracranial infection, etc.; non-surgery-related: respiratory and cardiovascular complications, etc.); ➃ length of hospital stay (postoperative hospital stay, total hospital stay); ➄ 30-days reoperation/rehospitalization rate and mortality rate.	N
5	Effects of preoperative oral carbohydrates on patients undergoing ESD surgery under general anesthesia: a randomized control study	Wang et al. (41)	China	Endoscopic submucosal dissection	73 cases (intervention group: 36; control group: 37)	Drink 710 mL of 5% carbohydrate solution (3% energy, 2% sodium) orally the night before the operation. Take 355 mL orally 2 h before the operation.	Fasting for 10 h before surgery	1. Subjective comfort: VAS scores of thirst, hunger, dry mouth, nausea, vomiting, fatigue before anesthesia induction, after extubation, and after returning to the ward (0–10 points); 2. Gastric related indicators: before anesthesia induction, gastric ultrasound score (grade 3), gastroscopic suction residual fluid volume, gastric peristalsis score (grade 4), surgical operation score (grade 4); 3. Clinical outcomes: time to first flatus, time to first water intake, duration of hemostatic drugs, length of hospital stay, and hospitalization expenses; 4. Complications: postoperative bleeding rate, fever rate (body temperature > 37.5 °C)	N
6	Effect of preoperative oral carbohydrate on insulin resistance in patients undergoing lumbar disc herniation surgery	Fang et al. (20)	China	Posterior lumbar decompression, interbody fusion and internal fixation, and posterior lumbar discectomy	94 cases (intervention group: 48; control group: 46)	Drink 300 mL of 14.2% carbohydrate solution orally 2 h before surgery (containing 14.2 g carbohydrate per 100 mL, with maltodextrin, crystalline fructose, glucose, etc.)	Fast from midnight on the day before surgery. Drink 300 mL of distilled water 2 h before surgery.	1. Metabolic and stress indicators: blood glucose (venous blood test), insulin concentration (venous blood test), insulin resistance index (HOMA-IR, calculated by the homeostasis model assessment method), C-reactive protein (CRP), white blood cells (WBC) at 3 h before surgery, the first day after surgery, and the third day after surgery; 2. Subjective comfort: thirst, hunger, and anxiety (VAS score, 0–10 points) at 1 h before surgery and 1 h after surgery; 3. Complications: incidence of intraoperative aspiration, postoperative nausea and vomiting, and postoperative abdominal distension.	N
8	Perioperative effects of preoperative oral carbohydrates in ERCP patients	Mutalif et al. (25)	China	Endoscopic retrograde cholangiopan creatography	132 cases (ERAS group: 69; control group: 63)	Drink 400 mL of 12.5% maltodextrin-fructose drink orally 2 h before surgery.	Fast and abstain from water for 6 h before surgery.	1. Subjective score: fatigue Scale-14 score at 2 h after operation, abdominal pain score at 2 h after operation (1–10 points); 2. Serological indicators: blood glucose (GLU), amylase (AMY), white blood cell (WBC) levels at 18 h after operation; 3. Complications: incidence of postoperative acute pancreatitis, acute cholangitis and bleeding; 4. Rehabilitation and economic indicators: postoperative hospital stay and hospitalization costs	N
9	The effects of preoperative oral carbohydrate on frequency of T and NK cells in patients with cervical cancer treated using neoadjuvant chemotherapy and surgery: a prospective cohort study	Zhang et al. (43)	China	Laparoscopy-assisted radical hysterectomy + bilateral salpingo-oophorectomy + pelvic lymph node dissection	77 cases (the non-NAC group: 26; the NAC group: 25; the NAC + CHO group: 26)	NAC + CHO group: drink 300 mL of ShuNeng orally 2 h before surgery.	Both the non-NAC group and NAC group: fast from solids for 8 h and from liquids for 4 h before surgery.	1. Main outcome: percentages of different lymphocyte subsets (NK cells, CD3+, CD4+, CD8+ cells) and the CD4+/CD8+ ratio (on the day of first admission, on the day before surgery, immediately after extubation, on the day after surgery); 2. Secondary outcome: baseline clinical data (age, BMI, operation time, intraoperative blood loss, fluid infusion volume)	N
10	The influence of preoperative oral administration of carbohydrates on the stress response, inflammatory index levels and gastrointestinal function recovery after laparoscopic cholecystectomy	He et al. (23)	China	Laparoscopic cholecystectomy	82 cases (intervention group: 41; control group: 41)	Drink 400 mL of the Su Qian 2 h before the operation.	Fast for 8 h before surgery and abstain from drinking from the night before surgery. (For surgeries scheduled between 9:00 and 11:00 a.m., the duration of no drinking is approximately 10–12 h.)	1. Inflammatory indicators: postoperative levels of C-reactive protein (CRP), interleukin-6 (IL-6), and tumor necrosis factor-α (TNF-α) (dual antibody sandwich enzyme-linked immunosorbent assay); 2. Stress response indicators: postoperative insulin (RI) level (radioimmunoassay), insulin resistance index (HOMA-IR, homeostasis model assessment); 3. Gastrointestinal function recovery indicators: postoperative time of abdominal distension relief, defecation, and bowel sounds.	N
11	Effects of preoperative oral carbohydrate on insulin resistance and psychological rehabilitation in patients after ERCP	Gong et al. (24)	China	Endoscopic retrograde cholangio pancreatography	120 cases (intervention group: 60; control group: 60)	Drink 1,000 mL of 10% glucose solution at 20:00 p.m before surgery, and drink 500 mL 2 h before surgery.	Fast for 8 h and withhold liquids for 6 h before surgery.	1. Intraoperative safety indicators: intraoperative aspiration; 2. Metabolic indicators: blood glucose, insulin and homeostasis model assessment of insulin resistance (HOMA-IR) during perioperative period (before operation, 3 h, 1 day and 3 days after operation); 3. Psychological and subjective evaluation indicators: postoperative self-rating anxiety scale (SAS) score, nursing satisfaction score, treatment compliance score, quality of life score (Karnofsky percentage method); 4. Rehabilitation and complications indicators: length of hospital stay, incidence of postoperative complications (pancreatitis, etc.)	N
12	Effects of preoperative oral single-dose and double-dose carbohydrates on insulin resistance in patients undergoing gastrectomy: a prospective randomized controlled trial	Chen et al. (38)	China	Radical gastrectomy for gastric cancer (including open/laparoscopic approach, distal gastrectomy/total gastrectomy, Billroth-I/II, Roux-en-Y digestive tract reconstruction)	136 cases (intervention group: 68; control group: 68)	Double-dose group: drink 1,000 mL of 10% glucose solution between 22:00 and 24:00 on the evening before surgery, and drink 500 mL 2–3 h before the surgery.	Single-dose group: drink 10% glucose solution orally 2–3 h before the surgery.	1. Main outcome: insulin resistance indicators (fasting blood glucose FPG, fasting insulin FIns, HOMA-IR, HOMA-IS, HOMA-β at the time of preoperative day 1, preoperative 3 h, and postoperative day 1); 2. Secondary outcomes: ➀ subjective comfort: VAS scores for thirst, hunger, etc., at preoperative day 1, preoperative 3 h, preoperative 1 h, and postoperative day 1; ➁ inflammatory mediators: CRP, IL-6, TNF-α at preoperative day 1, preoperative 3 h, and postoperative day 1; ➂ immune indicators: CD3^+^, CD4^+^, etc., at preoperative day 1, preoperative 3 h, and postoperative day 1; ➃ postoperative recovery: time of first defecation, time of fluid intake, length of hospital stay; ➄ complications: postoperative infection, anastomotic leakage, etc., 30-days unplanned readmission/reoperation rate; ➅ additional indicators: number of nocturnal urinations (≥2 times) before surgery, sleep time before surgery	N
13	Effect of preoperative oral glucose solution on the perioperative period of gastric cancer patients	Zhai et al. (27)	China	Gastric cancer surgery (including subtotal gastrectomy + lymph node dissection, total gastrectomy)	100 cases (intervention group: 50; control group: 50)	Drink 250 mL of 5% glucose solution at 22:00 1 day before surgery, and drink 250 mL of 5% glucose solution at 6:00 on the day of surgery.	Fast for 8–12 h before surgery.	1. Metabolic indicators: blood glucose levels before and 2 h after oral glucose administration and after operation (venous blood test); The insulin resistance index (HOMA-IR) was calculated before and after operation. 2. Subjective feelings: the incidence of preoperative thirst and hunger; 3. Safety indicators: incidence of aspiration, risk of reflux and aspiration (gastric volume/body weight ratio evaluation); 4. Rehabilitation indicators: postoperative exhaust time and hospital stay.	N
14	Effect of preoperative oral administration on safety and comfort in patients undergoing laparoscopic hysterectomy	Gu et al. (26)	China	Laparoscopic total hysterectomy	90 cases (group A: 30; group B: 30; group C: 30)	Group A: drink 300 mL of ShuNeng orally 3 h before surgery.	Group B: drink 200 mL of warm water 3 h before surgery. Group C: fast for 8 h before surgery.	1. Subjective comfort: thirst, hunger, anxiety before operation and anesthesia (VAS score, 0–10 points); (2) Metabolism-related indexes: blood glucose in the morning and on the first day after operation (measured by portable glucose meter), serum insulin concentration (measured by automatic chemiluminescence immunoassay analyzer), insulin resistance index (HOMA-IR), insulin sensitivity index (ISI); (3) Rehabilitation and complications indicators: incidence of postoperative nausea and vomiting, time to first anal exsufflation, postoperative hospital stay, intraoperative reflux and aspiration complications.	N
15	Preoperative carbohydrate loading and intraoperative goal-directed fluid therapy for elderly patients undergoing open gastrointestinal surgery: a prospective randomized controlled trial	Liu et al. (34)	China	Open gastrointestinal surgery (including gastrectomy, colectomy, and proctectomy)	112 cases (GDFT group: 55; CFT group: 57)	Drink 200 mL of Shuneng 2 h before the operation.	Fast for 8 h and abstain from drinking for 4 h before surgery.	1. Primary outcome: the incidence of postoperative complications (including infection, respiratory, cardiovascular and other types of complications); 2. Secondary outcomes: ➀ recovery of gastrointestinal function: time to first flatus and time to first oral feeding; ➁ hospitalization related: postoperative hospital stay, hospitalization cost, ICU admission rate, postoperative mortality; ➂ intraoperative indicators: fluid infusion volume (crystalloid, colloid), urine volume, estimated blood loss, vasoactive drug use; ➃ blood lactic acid levels: 5 min before anesthesia induction (T1), 60 min after operation (T2), and at the end of operation (T3).	N
16	Effects of preoperative carbohydrate intake on inflammatory markers and clinical outcomes in elderly patients undergoing radical prostatectomy: a single-center, double-blind randomized controlled trial	Hu et al. (45)	China	Radical prostatectomy	90 cases (CHO group: 28; placebo group: 30; fasting group 32)	Drink 800 mL of Su Qian from 19:00 to 24:00 on the evening before surgery, and drink 400 mL 2–3 h before surgery.	Placebo group: drink 800 mL of flavored water (containing sucralose and citric acid, 0 kcal/100 mL, osmolarity 107 mOsm/kg, pH 5.0) on the night before surgery, and drink 400 mL of the same flavored water 2–3 h before surgery. Fasting group: do not drink any liquid before surgery.	1. Primary outcome: the levels of inflammatory markers (IL-6, IL-8, IL-10, TNF, CRP) before operation and at 1, 3, and 7 days after operation; 2. Secondary outcomes: ➀ cellular immunity: CD3, CD4, CD8; ➁ comfort: VAS scores of anxiety, hunger and thirst before operation and at 1, 3 and 7 days after operation; ➂ grip strength index (grip strength/body weight × 100%); ➃ clinical outcomes: time to first flatus, time to stand independently after surgery, time to first drinking/eating, and incidence of infection on postoperative day 1, 3 and 7	N
17	Consequences of preoperative oral carbohydrate consumption in septal deviation patients undergoing endoscopic septoplasty: a retrospective cohort study	Zhu et al. (51)	China	Selective endoscopic septoplasty (endoscopic septoplasty)	337 cases (CHO group: 63; control group: 274)	Drink 800 mL of Su Qian between 20:00 and 22:00, and drink 400 mL 2 h before surgery, respectively.	Fast from solids for 12 h before surgery. Drink 800 mL of plain boiled water between 20:00 and 22:00, and drink 400 mL 2 h before surgery.	1. Primary outcome: risk of acute postoperative hypertension (defined as a ≥20% increase in postoperative systolic/diastolic blood pressure, including systolic, diastolic, and total APH); 2. Secondary outcomes: ➀ length of hospital stay; (2) hospitalization costs; ➂ sleep time of the night before surgery; ➃ the amount of infusion on the day of operation; ➄ incidence of postoperative nausea and vomiting; ➅ the incidence of aspiration	N
18	Effects of preoperative oral carbohydrate electrolyte drinks on preoperative hypokalemia incidence in patients scheduled for laparoscopic colorectal resection: a three-arm randomized clinical trial	Deng et al. (44)	China	Laparoscopic colorectal cancer resection (including colectomy and proctectomy);	122 cases (control group: 42; placebo group: 40; treatment group;40)	Drink 14.2% carbohydrate solution at a dose of 5–6 mL/kg based on body weight, 2–3 h before surgery.	Control group: fast from midnight before surgery and abstain from drinking. Placebo group: drink 14.2% carbohydrate clear solution at a dose of 5–6 mL/kg 2–3 h before surgery.	1. Primary outcome: the incidence and severity of preoperative hypokalemia (normal: 3.5–5.5 mmol/L; mild: 3.0–3.5 mmol/L; moderate: 2.5–3.0 mmol/L; severe: <2.5 mmol/L); 2. Secondary outcomes: ➀ preoperative comfort: thirst, hunger, anxiety, drink taste VAS score (0–10 points); ➁ postoperative rehabilitation: first exhaust/defecation time, first feeding time, ambulation time, postoperative hospital stay, hospitalization expenses; ➂ complications: postoperative nausea and vomiting, abdominal distension, arrhythmia, anastomotic leakage, etc.	N
19	Effects of preoperative oral electrolyte carbohydrate nutrition supplement on postoperative outcomes in elderly patients receiving total knee arthroplasty: a prospective randomized controlled trial	He et al. (40)	China	1. Unilateral total knee arthroplasty (TKA); 2. Surgical approach: standard medial parapatellar approach. Prosthesis type: cement-fixed 3. Depuy Attune knee prosthesis	94 cases (intervention group: 47; control group: 47)	Drink 250 mL of meal nutrition supplement orally 6 h before surgery, and drink 200 mL of electrolyte-carbohydrate nutrition supplement orally 2 h before surgery.	Drink 250 mL of dietary nutritional powder 6 h before surgery; (total energy: 300 kcal, protein: 12 g, fat: 3 g, carbohydrate: 56 g, sodium: 558 mg, potassium: 805 mg, calcium: 90 mg.) Drink 200 mL of water 2 h before surgery.	1. Primary outcome: patient-reported comfort indicators (PRCIs) - 6 VAS scores (0–10 points) for hunger, thirst, nausea, etc. 1 h before surgery and 1 day after surgery, anxiety score (APAIS scale) 1 day before surgery, and general comfort score (GCQ scale) 1 day after surgery; 2. Secondary outcomes: ➀ laboratory indicators: Electrolytes (Na^+^, K^+^, Ca^+^), hemoglobin, albumin, IL-6, CRP 1 day after surgery, blood glucose 2 h before surgery, 2 h after surgery, and 6 h after surgery; ➁ clinical indicators: perioperative vital signs, nutritional risk score (NRS2002), knee joint function score (HSS, 1 day before surgery, 1 month and 3 months after surgery), infusion volume, operation duration, blood loss, hospital stay, satisfaction; ➂ complications: aspiration, infection, thrombosis, etc., 30/60-days unplanned readmission rate and 30-day/1-year mortality rate	N
20	Effects of preoperative oral multidimensional carbohydrates on perioperative stress response in elderly patients undergoing total hip replacement	Wei et al. (28)	China	Unilateral total hip arthroplasty	88 cases (group O: 30; group C: 29; group W: 29)	Drink 5 mL/kg of ShuNeng orally 2 h before surgery.	Group C: fast for 6 h and withhold liquids for 8 h before surgery. Group W: drink 5 mL/kg of warm water orally 2 h before surgery.	1. Stress and metabolic parameters: blood glucose, adrenocorticotropic hormone (ACTH) and albumin concentrations (detected by automatic biochemical analyzer) before oral solution (T0), 10 min before anesthesia (T1), after awakening (T2), and the next morning after surgery (T3); 2. Subjective comfort: thirst and hunger before anesthesia and after recovery (VAS score, 0–10 points); 3. Complications: the incidence of perioperative reflux, aspiration, nausea and vomiting; 4. Vital signs: HR, MAP, body temperature and BIS values at 5 min after entering the room and immediately after intubation.	N
21	Efficacy of the oral administration of maltodextrin fructose before major abdominal surgery: a prospective, multicenter clinical study	Qin et al. (37)	China	Major abdominal surgerie (gastrectomy, colorectal resection, pancreatico duodenectomy)	223 cases (intervention group: 111; 112 control group: 112)	Drink 800 mL of Su Qian 10 h before surgery, and drink 400 mL orally 2 h before surgery.	Fast for 6 h before the operation. Drink 800 mL of water 10 h and 2 h before the operation respectively.	1. Primary outcome: Insulin resistance index, measured at baseline, 1 day after surgery, and 3 days after surgery (formula: fasting blood glucose × fasting insulin/22.5); 2. Secondary outcomes: ➀ metabolic indicators (fasting blood glucose, fasting insulin, HOMA-β, HOMA-ISI, at baseline, 1 day after surgery, and 3 days after surgery); ➁ subjective comfort (anxiety, thirst, appetite, nausea, fatigue VAS scores, at baseline and 1 h before surgery); ➂ intraoperative blood glucose (30/60/120/180 min after the start of the surgery); ➃ clinical outcomes (infection/non-infection complications, time to first flatus, hospital stay, incidence of aspiration during anesthesia).	Y
22	The effect of preoperative oral carbohydrate on the incidence of complications in PACU after general anesthesia: a prospective cohort study	Zhang et al. (49)	China	Open abdominal surgery or laparoscopic abdominal surgery (related to gastrointestinal or gynecological diseases)	307 cases (the POC group: 154; the control group153)	Drink 355 mL of Outfast orally 2 h before surgery.	Fast from midnight before the operation.	1. Primary outcome: total incidence of PACU complications (including delayed awakening, agitation upon awakening, hypoxemia, hypertension, hypotension, moderate to severe pain, nausea and vomiting, hypothermia, and chills); 2. Secondary outcomes: awakening time (from drug withdrawal to active response to verbal commands), extubation time (from drug withdrawal to extubation), PACU stay time.	N
23	Effects of preoperative oral enzyme-hydrolyzed rice flour solution on gastric emptying and insulin resistance in patients undergoing laparoscopic cholecystectomy: a prospective randomized controlled trial	Yang et al. (39)	China	Laparoscopic cholecystectomy	100 cases (intervention group: 50; control group: 50)	Drink 300 mL of EHR orally 2–3 h before the operation.	Drink 300 mL of clear water 2–3 h before the operation.	1. Main outcome: “gastric fullness” occurrence rate (T1: 2 h after the last administration; T2: re-measured 1 h after gastric fullness in those who were gastric full at T1; gastric fullness defined: solid contents or liquid volume > 1.5 mL/kg); 2. Secondary outcomes: ➀ gastric emptying-related: cross-sectional area of the gastric antrum in the semi-recumbent position/right lateral position at T1 (CSA), gastric volume (GV), GV/kg, Perlas classification; ➁ comfort: preoperative hunger, thirst, satisfaction VAS score (0–10 points); ➂ postoperative indicators: incidence of nausea and vomiting within 24 h, first defecation time, hospital stay, incidence of reflux and aspiration.	N
24	Application of preoperative oral carbohydrates in thyroid cancer surgery	Qian and Ke (18)	China	Radical thyroidectomy plus central lymph node dissection (subtotal and total thyroidectomy)	134 cases (intervention group: 70; control group: 64)	Drink 1,000 mL of 5% glucose solution 10 h before surgery, and drink 250 mL 2 h before surgery.	Fast for 10 h before surgery and withhold liquids for 4 h before surgery.	1. Rehabilitation indicators: postoperative first exhaust time, first defecation time, length of hospital stay, total cost of hospitalization; 2. Blood indicators: fasting blood glucose, white blood cell count, C-reactive protein level (fasting blood test) before operation and 1 day and 3 days after operation; 3. Complications: the incidence of aspiration during anesthesia induction, postoperative nausea and vomiting, numbness of hands and feet, bleeding asphyxia, hoarseness, choking on drinking, and chylous fistula.	N
25	Effects of preoperative carbohydrate beverage on gastric volume before anesthesia induction and perioperative hypotension in elderly patients undergoing thoracic surgery	Zhou et al. (15)	China	Thoracoscopic lobectomy	58 cases (intervention group: 29; control group: 29)	Drink 4 mL/kg of 5% glucose solution orally 2 h before induction of anesthesia.	Fast from midnight on the evening before surgery.	1. Gastric volume-related indicators: gastric antrum cross-sectional area (CSA), gastric volume (GV), gastric volume/body weight (GV/W) (measured by ultrasound to assess the risk of reflux and aspiration) in the semi-reclining position before anesthesia induction; 2. Vital signs: systolic blood pressure (SBP), mean arterial pressure (MAP) and heart rate (HR) from T1 (5 min after admission) to T7 (6 h after operation); 3. Vasoactive drugs use: the usage and dosage of ephedrine and norepinephrine during perioperative period; Postoperative indicators: the first postoperative exhaust time, the incidence of postoperative nausea and vomiting within 24 h, the postoperative hospital stay, the incidence of pulmonary complications; 5. Hypotensive related: incidence of perioperative SBP decrease >20% and lasting >30 s	N
26	Ultrasound assessment of gastric content in patients undergoing laparoscopic cholecystectomy after preoperative oral carbohydrates: a prospective, randomized controlled, double-blind study	Ge et al. (31)	China	Laparoscopic cholecystectomy, partially combined with laparoscopic exploration of the common bile duct	91 cases (CHO group: 46; control group: 45)	Drink 800 mL of Su Qian at 21:30 on the evening before surgery, and drink 400 mL from 5:00 to 5:30 on the day of surgery.	Fast from solid food after 20:00 and abstain from drinking after 21:30 before surgery.	1. Primary outcome: incidence of gastric fullness (defined as gastric solid or liquid contents >1.5 mL/kg), assessed at T1 (2 h) and T2 (3 h); Secondary outcomes were: ➀ gastric antrum cross-sectional area (CSA, semi-seated, right-lateral position); ➁ gastric volume (GV), gastric volume/body weight (GV/kg); ➂ Perlas grading (T1); ➃ preoperative comfort (hunger, thirst, satisfaction VAS score, 0–10 points); ➄ postoperative indicators (24 h incidence of nausea and vomiting, time to first flatus, operation time, incidence of reflux and aspiration).	N
27	Safety of high-carbohydrate fluid diet 2 h versus overnight fasting before non-emergency endoscopic retrograde cholangio pancreatography: a single-blind, multicenter, randomized controlled trial	Meng et al. (32)	China	Endoscopic retrograde cholangio pancreatography including therapeutic procedures such as sphincterotomy, balloon dilation, stone extraction, and pancreatic duct stenting.	1,292 cases (CFD group: 634; fasting group: 658)	CFD group: drink 400 mL of 12.5% maltodextrin solution 2 h before surgery.	Fast for 6 h before surgery.	1. Primary outcomes: ➀ postoperative fatigue score (4-h, 20-h, 14-point scale); ➁ postoperative abdominal pain score (4-h, 10-point scale); 2. Secondary outcomes: ➀ procedure related time (intubation time, total procedure time); ➁ complications (PEP, cholangitis, cholecystitis, etc.); ➂ metabolic indicators (intraoperative/2 h postoperative blood glucose, urinary ketone bodies); ➃ recovery time (time to resume eating, sitting up, and getting out of bed); ➄ the amount of residual fluid in stomach; ➅ length of hospital stay; ➆ late complications were followed up for 1 month	Y
28	Effect of preoperative oral carbohydrate on the postoperative recovery quality of patients undergoing daytime oral surgery: a randomized controlled trial	Tang et al. (47)	China	Oral and maxillofacial surgery (mainly impacted teeth extraction, a small number of other intraoral procedures)	92 cases (intervention group: 47; control group: 45)	Drink 4 mL/kg of Outfast orally according to body weight 2–3 h before surgery.	Fast after midnight.	1. Primary outcome: quality of recovery (QoR-15 score, 0–150) measured before and 24 h after surgery (including 5 dimensions of physical comfort and emotional state); 2. Secondary outcomes: ➀ patient comfort (NRS score, 0–10, including thirst, hunger and other 7 indicators, preoperative and postoperative 24 h); ➁ satisfaction (5-point scale, preoperative and postoperative 24 h); ➂ perioperative indicators (blood glucose at baseline, anesthesia induction, postoperative exhaust time); ➃ hemodynamics (MAP and HR at T0 baseline and 5 min after intubation at T1); ➄ adverse events (aspiration, readmission, etc.)	N
29	Effect of preoperative oral carbohydrates on insulin resistance in patients undergoing laparoscopic cholecystectomy: a randomized controlled trial	Wang et al. (46)	China	Laparoscopic cholecystectomy	129 cases (P1 group: 42; P2 group: 42; control group: 45)	Group P1: drink 200 mL of ShuNeng orally 2–4 h before surgery. Group P2: drink 400 mL of ShuNeng orally 2–4 h before surgery.	Fast after midnight before surgery.	1. Primary outcome: insulin resistance indicators (fasting plasma glucose (FPG), fasting insulin (FINS), and fasting glucagon at 1 day before T1, 10 min before T2 anesthesia, and 1 h after T3 surgery, HOMA-IR, HOMA-β, and HOMA-IS were calculated; 2. Secondary outcomes: ➀ gastric emptying assessment (gastric antrum cross-sectional area CSA, gastric volume GV, GV/body weight, Perlas classification); ➁ subjective comfort (VAS score of thirst and hunger at T2 and T3, 0–10); ➂ hemodynamics (heart rate and mean arterial pressure at admission, induction, skin incision and other time points)	N
30	Endoscopic assessment of gastric emptying in older adults after preoperative administration of 5% glucose solution: a randomized controlled study	Liu et al. (36)	China	Gastroscopy	100 cases (intervention group: 50; control group: 50)	Drink 5 mL/kg of 5% glucose solution orally according to body weight 2 h before surgery.	Fast after midnight before surgery.	1. Primary outcome: gastric volume (GV) measured by gastroscopic aspiration; Secondary outcomes: ➀ gastric volume/body weight (GV/kg); ➁ blood glucose (fasting, 0.5 h after medication and at discharge from PACU); ➂ comfort VAS score (hunger, thirst, anxiety); ➃ gastric mucosa clarity score (total score of four regions); ➄ adverse events (reflux, aspiration, abnormal circulation, etc.); ➅ factors affecting gastric volume (multiple linear regression analysis)	N
31	Application of 12.5% maltodextrin-fructose solution in patients with early gastric cancer undergoing endoscopic submucosal dissection	Zhuo et al. (53)	China	Endoscopic submucosal dissection	116 cases (intervention group: 57; control group: 59)	Drink 800 mL of ShuNeng orally 8 h before surgery, and drink 400 mL orally 2 h before surgery.	Fast for 8 h and abstain from liquids for 4 h before surgery.	1. Blood biochemical and inflammatory indicators: fasting blood glucose (FBG), insulin, electrolytes (potassium, sodium, etc.), total protein (TP), albumin (Alb) and other nutritional indicators, C-reactive protein (CRP), interleukin-6 (IL-6) and other inflammatory factors before operation, on the first day and the third day after operation; 2. Subjective comfort: hunger, thirst, pain, anxiety before operation and 24 h after operation (VAS score, 0–10 points); 3. Post-traumatic growth: preoperative and postoperative post-traumatic growth inventory (PTGI) scores; 4. Rehabilitation and economic indicators: length of stay, hospitalization expenses; 5. Complications: the incidence of perioperative reflux and aspiration, postoperative bleeding, nausea and vomiting, abdominal distension, abdominal pain, etc.	N
32	Effects of preoperative oral carbohydrate intake on postoperative insulin resistance and perioperative safety in elderly patients with type 2 diabetes undergoing joint replacement	Yan et al. (21)	China	Unilateral joint replacement (including hip replacement, knee replacement, femoral head replacement, etc.)	90 cases (OC1 group: 30; OC2 group: 30; control group: 30)	OC1 group: drink 100 mL of Su Qian orally 2 h before surgery. OC2 group: drink 200 mL of Su Qian orally 2 h before surgery.	Drink 0 mL of Su Qian orally 2 h before surgery.	1. Gastric volume related indicators: before administration (T0), immediately after administration (T1), before anesthesia induction (T2), gastric antrum cross-sectional area (CSA), gastric volume (GV), gastric volume per body weight (GV/W) (ultrasound detection); 2. Vital signs: heart rate (HR), mean arterial pressure (MAP), body temperature and bispectral index at 5 min after entering the operating room, 1 h after the beginning of the operation and at the time of extubation; 3. Peripheral blood indexes: blood glucose, adrenocorticotropic hormone (ACTH) and albumin before administration, before anesthesia induction, anesthesia recovery, and the next morning; Fasting insulin (FINS) and insulin resistance index (HOMA-IR) were measured before and 3 days after operation. 4. Clinical symptom score: the scores of anxiety, depression, hunger, thirst, pain, nausea and fatigue before operation and 24 h after operation (0–6 points, four grades); 5. Postoperative and complication indicators: incidence of postoperative nausea and vomiting, incidence of infection, time to first exhaust, time to start eating, length of hospital stay.	N
33	Safety of preoperative carbohydrate nutrient solution in anesthesia for elderly patients with intertrochanteric fracture	Wu et al. (17)	China	Intertrochanteric fracture surgery	48 cases (intervention group: 24; control group: 24)	Drink 200 mL of Junbeian orally 2 h before surgery.	Fast for 8 h before surgery.	1. Subjective status: the incidence of symptoms such as thirst, palpitation and fear 30 min before surgery; The satisfaction of anesthesia and the incidence of adverse reactions such as nausea and vomiting within 24 h after operation; 2. Gastric emptying related: gastric residual volume before anesthesia (calculated by the cross-sectional area of gastric antrum detected by ultrasound); 3. Intraoperative indicators: anesthesia time, blood gas status (pH, PaCO2, HCO3, etc.), heart rate/blood pressure fluctuation, aspiration/vomiting; 3. 4. Glucose metabolism related: fasting blood glucose, serum insulin, insulin resistance index (HOMA-IR) at admission and during operation.	N
34	Effects of preoperative oral carbohydrates on insulin resistance and postoperative recovery in diabetic patients undergoing coronary artery bypass grafting: a preliminary prospective, single-blinded, randomized controlled trial	Zhang et al. (42)	China	Off-pump coronary artery bypass grafting surgery	62 cases (intervention group: 31; control group: 31)	Drink 355 mL of Outfast from 20:00 to 24:00 on the night before surgery.	Traditional fasting protocol: fast after 20:00 on the evening before surgery.	1. Primary outcome: insulin resistance indicators (HOMA-IR at admission, immediately after surgery, and on days 1/2/3/5 after surgery); 2. Secondary outcomes: ➀ inflammatory mediators (IL-1, IL-6, IL-8, IL-10, TNF-α, etc., on the 1st day after surgery); ➁ stress response (serum cortisol level on the 1st day after surgery); 3. Exploratory outcomes: hospital clinical outcomes (volume of thoracic drainage fluid, duration of mechanical ventilation, ICU stay time, total hospital stay time, incidence of complications, etc.)	N
35	Gastric residual volume, safety, and effectiveness of drinking 250 mL of glucose solution 2–3 h before surgery in gastric cancer patients: a multicenter, single-blind, randomized–controlled trial	Yang et al. (35)	China	Radical gastrectomy (open, laparoscopic, robotic surgery)	83 cases (intervention group: 42; control group: 41)	Drink 250 mL of 5% glucose solution orally 2–3 h before surgery.	Fast for 6–8 h before surgery.	1. Primary outcome: preoperative gastric residual volume (measured by aspiration under gastroscope after tracheal intubation); 2. Secondary outcomes: ➀ intragastric pH (Delta 320 pH monitor); ➁ preoperative comfort (thirst and hunger VAS score, 0–10 points); ➂ insulin sensitivity (QUICKI index, before operation, immediately after operation, 1/3/5 days after operation); ➃ surgical indicators (operation time, intraoperative blood loss, infusion volume, etc.); ➄ postoperative rehabilitation (time of first flatus/defecation/fluid diet); ➅ postoperative complications (including abdominal infection and anastomotic leakage within 30 days); ➆ length of hospital stay and 30-days readmission rate.	Y
36	Effect of preoperative oral carbohydrate on postoperative delirium in elderly patients undergoing lower extremity orthopedic surgery: a prospective randomized trial	Li et al. (50)	China	Lower extremity orthopedic surgery (hip or knee replacement)	80 cases (intervention group: 40; control group: 40)	Drink 200 mL of Fuan orally 2 h before surgery.	Fast after midnight before surgery.	1. Primary outcome: incidence of postoperative delirium (POD) assessed by 3D-CAM scale from postoperative days 1–5; 2. Laboratory indicators: fasting blood glucose, interleukin-6 (IL-6) and C-reactive protein (CRP) levels (detected by ELISA) before operation and at 1, 3 and 5 days after operation; 3. Vital signs: mean arterial pressure (MAP) and heart rate (HR) at 5 min after entering the operating room (T0), 5 min after anesthesia (T1), 30 min after operation (T2) and at the end of operation (T3); 4. Postoperative complications: incidence of thirst, nausea and vomiting	N
37	The role of oral carbohydrate beverage before anesthesia in enhanced recovery after surgery in obese patients	Liu et al. (30)	China	54 patients underwent gastrointestinal surgery and 30 patients underwent urologic surgery.	84 cases (carbohydrate group: 26; pure water group: 28; control group: 30)	Carbohydrate group: drink 250 mL of ShuNeng orally 2 h before anesthesia.	Pure water group: drink 250 mL of pure water orally before anesthesia. Control group: fast for 8 h before surgery and do not take any oral liquids.	1. Gastric volume related: gastric volume (GV) at T1, T2 and T3, gastric volume/body weight (GV/W) (monitored by ultrasound); 2. Subjective feelings: self-rating anxiety scale (SAS) score, visual analogue scale (VAS) hunger and thirst score at T3; 3. Quality of postoperative recovery: quality of recovery scale (QoR-40) score at 24 h after operation (including five dimensions of emotional state, physical comfort, self-care ability, psychological support and pain); 4. Complications: occurrence of reflux and aspiration during perioperative period, incidence of nausea and vomiting within 24 h after operation	N
38	Clinical value of preoperative oral carbohydrate loading in patients with diabetes: a cross-sectional study	Wang et al. (52)	China	Non-cardiac surgery (including gynecology, thyroid and breast surgery, urology surgery, thoracic surgery, hepatobiliary and pancreatic surgery, etc.)	410 cases (intervention group: 205; control group: 205)	Drink 400 mL of carbohydrate drink between 21:00 and 23:00 on the evening before surgery, and drink 200 mL orally 2 h before surgery.	Traditional preoperative fasting and water deprivation	1. Primary outcome: postoperative blood glucose (postprandial blood glucose 3 times a day, CV%); 2. Secondary outcomes: ➀ postoperative complications (pulmonary infection); ➁ length of hospital stay; ➂ hospitalization expenses; ➃ insulin use (preoperative insulin continuation, new insulin, continuous insulin infusion); ➄ ICU admission rate	N
39	Effect of preoperative carbohydrate intake on gastrointestinal function in elderly trauma patients under spinal anesthesia	Sun et al. (16)	China	Proximal femoral nail internal fixation and femoral head replacement were performed	81 cases (intervention group: 40; control group: 41)	Drink 200 mL of Mizone orally 2 h before surgery.	Fast at 22:00 before surgery.	1. Primary outcome: time to first flatus after surgery; Intestinal barrier function indexes (diamine oxidase, D-lactic acid, bacterial endotoxin, preoperative and postoperative detection); 2. Secondary indicators: ➀ operation related: operation time, intraoperative blood loss; ➁ vital signs: mean arterial pressure (MAP) and heart rate (HR) after entering the room (T1), after anesthesia (T2), at the midpoint of the operation (T3), at the time of leaving the room (T4); ➂ metabolism related: blood glucose level and its change rate before, T1, T4 and after operation, insulin resistance index (HOMA-IR) and its change rate before and after operation, C-reactive protein (CRP) and its change rate before and after operation; ➃ scale scores: self-rating anxiety scale (SAS), visual analogue scale (VAS), Ramsay sedation score before operation, 1 day and 2 days after operation; ➄ cognitive status: preoperative and postoperative delirium diagnostic scale (3D-CAM) was used to evaluate the incidence of delirium	N
40	The effect of preoperative oral carbohydrate on the perioperative period of fibula free flap surgery in patients with oral cancer: a retrospective study	Liu et al. (48)	China	Extended resection of oral cancer and simultaneous reconstruction with free fibular flap	87 cases (intervention group: 43; control group: 44)	Drink 800 mL of Dongze Sutang orally 10 h before surgery, and drink 200 mL orally 2 h before surgery.	Fast from solid food for 12 h and from water for 4 h before surgery.	1. Preoperative: VAS score of comfort (thirst, hunger, anxiety and sleep quality at 1 day and 1 h before surgery, 0–10 points); 2. Intraoperative: duration of operation, intraoperative blood loss, and infusion volume; 3. Postoperative: ➀ general situation (extubation time, hospitalization time, total cost); ➁ complications (flap crisis, wound infection, bleeding/hematoma, thrombosis, gastrointestinal reaction); ➂ laboratory indexes (hemoglobin, albumin, prealbumin, blood glucose on the first day of admission and the first day after operation, blood glucose 1 h before operation, PCT, IL-6 on the first day after operation)	N
41	The influence of oral carbohydrate solution intake on stress response before total hip replacement surgery during epidural and general anesthesia	Çeliksular et al. (61)	Türkiye	Total hip replacement was performed	80 cases (group G: 20; group GN: 20; group E: 20; group EN: 20)	GN group, EN group: drink 800 mL of PreopQ orally 8 h before surgery, and drink 400 mL orally 2 h before surgery.	Group G, group E: fast for 8 h before surgery.	1. Main outcome: Surgical stress response (blood glucose, insulin, cortisol, IL-6, testing time points: 1 day before surgery, preoperative preparation room, 5 min/1/2/6/24 h after incision); 2. Secondary outcomes: ➀ vital signs (heart rate, mean arterial pressure); ➁ postoperative pain VAS score (6/24 h after incision); ➂ incidence of nausea and vomiting	N
42	Does preoperative oral carbohydrate treatment reduce the postoperative surgical stress response in lumbar disc surgery?	Dilmen et al. (62)	Türkiye	Microscopic lumbar intervertebral disc resection and laminectomy	40 cases (intervention group: 20; control group: 20)	Drink 800 mL of Nutricia Preop orally from 21:00 to 24:00 on the evening before surgery; drink 400 mL orally 2 h before surgery.	Fast for 8 h before surgery.	1. Main outcome: surgical stress response and insulin resistance (blood glucose, plasma insulin, cortisol, interleukin - 6 IL-6, testing time points: 1 day before surgery, before anesthesia induction, after incision, 1/2/6/24 h after incision); 2. Secondary outcome: incidence of postoperative nausea and vomiting (PONV)	N
43	Do preoperative oral carbohydrates improve postoperative outcomes in patients undergoing coronary artery bypass grafts?	Şavluk et al. (63)	Türkiye	Coronary artery bypass grafting surgery	152 cases (Group 1: 38; Group 2: 37; Group 3: 38; Control group: 39)	Group 1: drink 800 mL of Nutricia orally 8 h before surgery; drink 400 mL orally 2 h before surgery. Group 2: drink 400 mL of Nutricia orally 8 h before surgery. Group 3: drink 400 mL of Nutricia orally 2 h before surgery.	Fast for 8 h before surgery.	1. Primary outcome: postoperative insulin requirement (an indicator of compensatory insulin resistance, insulin injection as needed when blood glucose is >180 mg/dL); 2. Secondary outcomes: ➀ use of positive inotropic drugs/vasopressors; ➁ mechanical ventilation time; ➂ ICU hospitalization time; ➃ preoperative comfort (VAS score: dry mouth, hunger, anxiety, nausea, 1–10 points); ➄ incidence of postoperative bleeding and arrhythmia.	N
44	The effect of preoperative oral carbohydrate solution intake on patient comfort: a randomized controlled study	Çakar et al. (64)	Türkiye	Thyroidectomy	90 cases (carbohydrate group: 30; 5% glucose group: 30; fasting group: 30)	Carbohydrate group: before the operation, orally take 800 mL of PreOp-Nutricia at midnight. Take 400 mL orally 2 h before the operation.	5% glucose group: infuse 1,000 mL of 5% glucose solution intravenously from midnight to 2 h before surgery. Fasting group: fast after midnight before surgery.	1. Primary outcome: preoperative discomfort (hunger, thirst, dry mouth, chills, headache, etc., VAS score, 0–10 cm); 2. Secondary outcomes: ➀ postoperative complications (nausea, vomiting, pain, VAS score); ➁ perioperative vital signs (blood pressure, pulse, body temperature); ➂ perioperative blood glucose levels.	N
45	The effect of preoperative oral carbohydrate administration on insulin resistance and comfort level in patients undergoing surgery	Onalan et al. (65)	Türkiye	Laparoscopic cholecystectomy	50 cases (OCS group: 25; control group: 25)	Drink 800 mL of Nutricia preop orally at midnight before surgery; drink 400 mL orally 2 h before surgery.	Fast after midnight before surgery.	Primary outcome: ➀ insulin resistance (HOMA-IR, normal < 2.7, based on both glucose and insulin levels); ➁ comfort (VAS score: hunger, thirst, anxiety, pain; GCS score: relief, relaxation, transcendence); Secondary outcomes: blood glucose and insulin levels at different time points (baseline, 2 h before surgery, and 1/3 h after surgery).	N
46	The effect of preoperative oral intake of liquid carbohydrate on postoperative stress parameters in patients undergoing laparoscopic cholecystectomy: an experimental study	Gümüs et al. (66)	Türkiye	Laparoscopic cholecystectomy	68 cases (intervention group: 35; control group: 33)	Drink 400 mL of 12.5% maltodextrin orally 2 h before surgery.	Fast for 12 h before surgery.	1. Primary outcome: postoperative stress parameters [blood glucose, insulin resistance (HOMA-IR), cortisol, norepinephrine, epinephrine]; 2. Detection time points: 2 h before surgery, 2 h after surgery, 24 h after surgery.	N
47	Optimizing postoperative clinical outcomes in spinal surgery through preoperative oral carbohydrate loading: a case-control study	İbrahimoğlu et al. (67)	Türkiye	Spinal surgery with transpedicular fixation	92 cases (intervention group: 46; control group: 46)	Drink 800 mL of 53% carbohydrate solution orally at midnight before surgery.	Fast after midnight before surgery.	1. Primary outcome: postoperative clinical outcomes (nausea and vomiting, use of antiemetics/analgesics, local inflammation, intraoperative/postoperative bleeding, time to first flatus/defecation, time to oral intake, time to first ambulation, and length of hospital stay); 2. Secondary outcomes: incidence of adverse events and complications within 24 h after surgery.	N
48	Effects of preoperative carbohydrates drinks on immediate postoperative outcome after day care laparoscopic cholecystectomy	Singh et al. (54)	India	Laparoscopic cholecystectomy	120 cases: (carbohydrate group: 40; placebo group: 40; control group: 40)	Intervention group: drink 400 mL of 12.5% carbohydrate orally from 8 to 10 p.m. on the day before surgery; drink 200 mL orally 2 h before surgery.	Placebo group: drink 400 mL of placebo orally from 8 to 10 p.m. the night before surgery; drink 200 mL orally 2 h before surgery. Control group: fast after midnight before surgery.	1. Primary outcome: postoperative nausea and vomiting (PONV) (incidence and frequency of occurrence within 0–4, 4–12, and 12–24 h); 2. Secondary outcome: postoperative pain (VAS score, 0–4, 4–12, and 12–24 h)	N
49	Effect of preoperative carbohydrate drink and postoperative chewing gum on postoperative nausea and vomiting in patients undergoing day care laparoscopic cholecystectomy: a randomized controlled trial	Balabolu et al. (55)	India	Laparoscopic cholecystectomy	100 cases (intervention group: 50; control group: 50)	Drink 200 mL of 12.5% carbohydrate beverage orally 2 h before surgery.	Fast after midnight before surgery.	1. Primary outcome: postoperative nausea and vomiting (PONV) (incidence, severity VAS score, dosage of rescue antiemetic drugs, PACU monitoring within 0–6 h after surgery, 48-h follow-up); 2. Secondary outcomes: ➀ postoperative pain (VAS score, dosage of rescue analgesic drugs); ➁ quality of recovery score (QoR-15 questionnaire, 6/24/48 h after surgery); ➂ intraoperative indicators (operation duration, anesthesia duration, duration of carbon dioxide pneumoperitoneum).	N
50	Randomized controlled trial comparing the effects of preoperative carbohydrate and non-carbohydrate loading on gastric emptying in diabetic and non-diabetic patients posted for elective surgery	Vishak et al. (56)	India	Inguinal hernia surgery	240 cases (DC group: 60; DS group: 60; NDC group: 60; NDS group: 60)	DS group, NDS group: drink 400 mL of solution containing 50 g of glucose orally 2 h before surgery.	DC group, NDC group: Drink 400 mL of water orally 2 h before surgery.	1. Main outcome: gastric volume (ultrasound qualitative grading + quantitative calculation, measured in the lateral position); 2. Secondary outcomes: ➀ capillary blood glucose (before surgery, 2 h after surgery, 6 h after surgery); ➁ Degree of thirst and discomfort (5-point Likert scale, 0 = least severe, 5 = most severe)	N
51	Effect of preoperative oral carbohydrate intake on perioperative hyperglycemia in Indian patients undergoing hip fracture fixation	Minz et al. (57)	India	Hip fracture fixation surgery (including reduction and fixation of intertrochanteric fractures of the femur, femoral neck fractures, as well as fixation with cortical and cancellous bone screws/dynamic hip screws, and joint replacement)	60 cases (intervention group: 30; control group: 30)	Drink 200 mL of Aptonia hydrated powder orally 2 h before surgery (containing approximately 24 g of carbohydrates).	Fast for 6 h before surgery.	1. Main outcome: perioperative blood glucose levels [measurement time points: at the time of CSE administration (T0), immediately after surgery (T1), and 24 h after surgery (T2)]; 2. Secondary outcomes: ➀ incidence of postoperative hyperglycemia (blood glucose > 180 mg/dL); ➁ insulin and blood urea levels; ➂ comfort level (hunger score, thirst score, incidence of anxiety, measured at T0 and T1); ➃ length of hospital stay, 30-days readmission rate, 3-months mortality rate	N
52	Effect of preoperative oral carbohydrate loading versus oral rehydration solution on enhanced recovery after surgery in elective open gynecological surgeries: a prospective interventional study	Jaiswal et al. (58)	India	Abdominal gynecological surgery	105 cases: (CHO group: 35; ORS group: 35; mineral water group: 35)	CHO group: drink 600 mL of commercial beverage (containing 75 g of carbohydrates) orally 10 h before surgery; drink 400 mL orally 3 h before surgery (containing 50 g of carbohydrates).	ORS group: drink 600 mL of rehydration solution (containing 8.1 g of carbohydrates) orally 10 h before surgery; drink 300 mL orally 3 h before surgery (containing 5.4 g of carbohydrates). Mineral water group: drink 600 mL of mineral water orally 10 h before surgery; drink 400 mL orally 3 h before surgery.	1. Main outcomes: ➀ subjective comfort level (VAS score: hunger, thirst, anxiety, nausea, fatigue, measured before and after administration); ➁ postoperative blood glucose (1 h after anesthesia, 24 h after); ➂ quality of recovery score (QoR-40, 24 h after surgery); 2. Secondary outcomes: ➀ gastric residual volume (preoperative ultrasound measurement); ➁ time for intestinal function recovery (first defecation time); ➂ hospital stay; ➂ postoperative complications (nausea, vomiting, surgical site infection, etc.)	N
53	Preoperative carbohydrate loading reduces perioperative insulin resistance and hastens functional recovery of remnant liver after living donor hepatectomy: an open-label randomized controlled trial	Kumar et al. (59)	India	Living donor liver resection surgery	70 cases (intervention group: 35; control group: 35)	Drink 400 mL of 12.5% maltodextrin solution orally on the evening before surgery; drink 200 mL orally 2 h before surgery.	Fast for 6 h before surgery.	1. Primary outcome: perioperative insulin resistance (PIR), evaluated using HOMA-IR (on the second day after surgery); 2. Secondary outcomes: ➀ residual liver function recovery (serum bilirubin, INR normalization time); ➁ postoperative nausea and vomiting (PONV, within 72 h after surgery); ➂ inflammatory markers (IL-6, CRP, from baseline to 7 days after surgery); ➃ hospital stay; ➄ postoperative complications (Clavien-Dindo classification); ➅ time to tolerate soft food.	N
54	Clinical and biochemical effects of preoperative oral carbohydrate loading versus fasting in major abdominal surgeries: a randomized clinical study	Nanda Lakshmi et al. (60)	India	Major abdominal surgeries (including pancreatic jejunostomy side-to-side anastomosis and colorectal surgeries)	54 cases (intervention group: 27; control group: 27)	Drink 400 mL of 12.5% maltodextrin solution orally at 22:00 on the evening before surgery; drink 200 mL orally 2 h before surgery.	Fast for 8 h and restrict fluids for 2 h before surgery.	1. Main outcome: perioperative changes in blood glucose (measurement time points: 1 day before surgery, 2 h before surgery, 6 h after surgery, 6 o’clock on the second day after surgery, 6 o’clock on the third day after surgery); 2. Secondary outcomes: ➀ perioperative albumin levels (1 day before surgery, 6 o’clock on the third day after surgery); ➁ subjective comfort (VAS score: fatigue, thirst, hunger, nausea, vomiting, measurement time: before anesthesia induction, 4/8/12/24 h after surgery); ➂ time to resume oral intake after surgery; ➃ postoperative hospital stay.	N
55	Evaluation of preoperative oral carbohydrate administration on insulin resistance in off-pump coronary artery bypass patients a randomized trial	Lee et al. (68)	South Korea	Multi-vessel off-pump coronary artery bypass grafting surgery	57 cases (intervention group: 28; control group: 29)	Drink 400 mL of NO-NPO orally from 21:00 to 23:00 on the evening before surgery; drink 400 mL orally 2–3 h before surgery.	Fast after midnight before surgery.	1. Main outcome: insulin sensitivity (measured by the short-term insulin tolerance test as the KITT value), measured after anesthesia induction and within 1 h after surgery; 2. Secondary outcomes: ➀ free fatty acid (FFA) concentration (before induction before surgery, immediately after surgery, and at 24/48 h); ➁ myocardial injury markers (peak of creatine kinase isoenzyme CK-MB); ➂ glycemic variability (coefficient of variation during surgery and 48 h after surgery); ➃ incidence of combined complications (stroke, acute kidney injury, etc.); ➄ ICU and hospital stay.	N
56	Effects of preoperative oral carbohydrate administration on patient well-being and satisfaction in thyroid surgery	Doo et al. (69)	South Korea	Open thyroid surgery	50 cases (intervention group: 25; control group: 25)	Drink 400 mL of NO-NPO orally 2 h before surgery.	Fast after midnight before surgery.	1. Primary outcome: ➀ patient comfort (seven indicators: thirst, hunger, dry mouth, etc., NRS 0–10), measured before and 6 h after surgery; ➁ patient satisfaction (5-point scale: 1 = very dissatisfied to 5 = very satisfied) was measured before operation and 6 h after operation; Secondary outcomes: ➀ Oral Schirmer test (salivary output, mm/5 min); ➁ blood glucose concentration (measured at 6 time points) and coefficient of variation; ➂ actual fasting time.	N
57	Effects of preoperative oral carbohydrates on quality of recovery in laparoscopic cholecystectomy: a randomized, double blind, placebo-controlled trial	Lee et al. (70)	South Korea	Laparoscopic cholecystectomy	139 cases (NO-NPO group: 46; placebo group: 44; MN-NPO group: 49)	NO-NPO group: drink 400 mL of NO-NPO orally from 8:00 to 10:00 on the evening before surgery; drink 400 mL orally 2 h before surgery.	Placebo group: drink 400 mL of placebo orally from 8:00 to 10:00 on the evening before surgery; drink 400 mL orally 2 h before surgery. MN-NPO group: fast after midnight before surgery.	1. Primary outcome: quality of recovery (QoR-40 questionnaire), covering 5 dimensions including emotional state, physical comfort, and psychological support, measured within 2 days before surgery and 1 day after surgery; 2. Secondary outcome: intraoperative hemodynamics (heart rate, mean arterial pressure, recorded at 9 time points); 3. Others: operation time, intraoperative blood loss, usage of analgesics and antiemetics within 24 h after surgery, complications (aspiration, pneumonia, etc.).	N
58	Preoperative intake of carbohydrate-rich drinks is associated with postoperative pulmonary complications in patients after gastric cancer surgery	Park et al. (71)	South Korea	Radical surgery for gastric cancer	142 cases (CRD group: 70; NPO group: 72)	CRD group: drink 400 mL of NO-NPO orally at 6:00 p.m. on the day before surgery.	NPO group: fast after midnight before surgery.	1. Main outcome: incidence of postoperative pulmonary complications (such as pneumonia, empyema, pneumothorax, etc., diagnosed through chest X-ray/CT); 2. Secondary outcomes: ➀ nutritional indicators (serum protein, albumin, blood sugar, cholesterol, measured before surgery and on the 1st/3rd/5th days after surgery); ➁ postoperative complications (anastomotic leakage, abdominal abscess, etc.); ➂ Clavien-Dindo complication grading; ➃ operation time, hospital stay.	N
59	Preoperative carbohydrate drink intake increases glycemic variability in patients with type 2 diabetes mellitus in total joint arthroplasty: a prospective randomized trial	Lee et al. (72)	South Korea	Total hip replacement surgery, total knee replacement surgery	46 cases (intervention group: 22; control group: 24)	Drink 400 mL of NO-NPO for 2–3 h before surgery.	Fast after midnight before surgery.	1. Main outcomes: ➀ glycemic variability (coefficient of variation, J index), measured at baseline, before surgery, immediately after surgery, and 1 h after surgery; ➁ insulin resistance (HOMA-IR), measured before surgery; 2. Secondary outcomes: ➀ gastric volume (measured by ultrasound, assessed before surgery); ➁ hormone/metabolic indicators (insulin, C-peptide, free fatty acids, etc.); ➂ postoperative complications (nausea/vomiting, delirium, wound dehiscence, etc.); ➃ hospital stay, intraoperative blood loss, pain score (VNRS).	N
60	The safety and effect of preoperative reduced fasting time by oral clear liquid administration in adult surgery patients: a randomized controlled trial	Lee et al. (73)	South Korea	Endoscopic complete extraperitoneal repair	60 cases (intervention group: 30; control group: 30)	Drink NO-NPO freely 2 h before surgery, with a mean intake of 520 ± 255 mL.	Fast after midnight before surgery.	1. Main outcomes: ➀ volume and pH of gastric contents (measured by nasogastric tube suction after intubation); ➁ preoperative/postoperative VAS scores for thirst and hunger (0–10 points); 2. Secondary outcomes: postoperative recovery room indicators (hoarseness of voice, incidence of nausea and vomiting, blood oxygen saturation SpO2); ➂ hospital stay, operation time.	N
61	Fast-track- recovery surgery with a whey protein-infused carbohydrate-loading drink pre-operatively and early oral feeding post operatively among surgical gynecological cancer patients: study protocol of an open labeled, randomized controlled trial	Ho et al. (74)	Malaysia	Gynecological cancer surgery	110 cases (intervention group: 55; control group: 55)	Drink 474 mL of Resource^®^ Breeze orally on the night before surgery; drink 237 mL orally 3 h before surgery.	Fast after midnight before surgery.	1. Main outcomes: ➀ postoperative hospital stay; ➁ clear fluid tolerance time; ➂ solid food tolerance time; ➃ intestinal function recovery time (intestinal gas release/defecation recovery); 2. Secondary outcomes: ➀ postoperative complications (nausea, vomiting, intestinal obstruction, infection); ➁ rehospitalization rate and reasons within 1 month after surgery; ➂ nutritional status (weight, muscle mass, upper arm circumference); ➃ biochemical indicators (hemoglobin, C-reactive protein, albumin, blood glucose); ➄ functional status (handgrip strength).	N
62	The effect of pre-endoscopy maltodextrin beverage on gastric residual volume and patient’s well-being: a randomized controlled trial	Zulkifli et al. (75)	Malaysia	Esophagogas troduodenoscopy	78 cases (intervention group: 38; control group: 40)	Drink 400 mL of Resource^®^ Breeze orally 2 h before surgery.	Drink 400 mL of plain water orally 2 h before surgery.	1. Main outcomes: ➀ gastric residual volume (GRV), measured directly by endoscopy through aspiration; ➁ patient comfort VAS score (including 5 items: hunger, thirst, anxiety, fatigue, and overall discomfort, with 0 indicating no discomfort and 100 indicating the most severe discomfort); 2. Secondary outcomes: safety (complications related to endoscopy, such as aspiration pneumonia, etc.)	N
63	A feasibility study on preoperative carbohydrate loading in older patients undergoing hip fracture surgery	Yap et al. (76)	Malaysia	Hip fracture-related surgeries	29 cases (intervention group: 12; control group: 17)	Drink 237 mL of Resource^®^ Breeze orally at 16:00 and 20:00 on the day before surgery, and drink 237 mL orally 2–6 h before surgery.	Avoid solid food for 6 h and refrain from oral intake for 2 h before surgery.	1. Main outcomes (feasibility indicators): ➀ recruitment rate (3–4 cases per month); ➁ dropout rate (29%); ➂ compliance (100%); ➃ tolerance (median VAS score of 10, with 10 being extremely tolerable); ➄ safety (no adverse events); 2. Secondary outcomes: ➀ incidence of postoperative nausea and vomiting; ➁ pain score (rest/active VAS); ➂ fatigue degree (VAS); ➃ muscle strength (grip strength, mid-thigh circumference of the non-surgical side); ➄ postoperative infection rate; ➅ hospital stay.	N
64	Kelulut honey as an alternate source of carbo-loading in abdominal surgery involving the digestive system: a randomized blinded comparative study	Krishnan et al. (77)	Malaysia	Scheduled abdominal surgeries related to the digestive system (including gallbladder surgeries, hernia surgeries, small intestine surgeries, colon surgeries, etc.)	63 cases (intervention group: 32; control group: 31)	Drink 800 mL of Kelulut honey producer orally 8 h before surgery (containing 135 g Kelulut honey, providing 94.5 g carbohydrates and 392 kcal energy); drink 400 mL orally 2 h before surgery (containing 68 g Kelulut honey, providing 47.3 g carbohydrates and 197 kcal energy).	Drink 800 mL of Resource^®^ Breeze orally 8 h before surgery; drink 400 mL orally 2 h before surgery.	1. Main outcomes: ➀ insulin resistance (blood glucose monitoring at multiple time points before and after the surgery); ➁ gastric residual volume (RGV, measured by nasogastric tube aspiration after anesthesia induction); 2. Secondary outcomes: ➀ postoperative complications (classified according to Clavien-Dindo scale); ➁ hospital stay; ➂ pain control (requirement for additional analgesia); ➃ functional recovery (time of gas expulsion, time of getting out of bed for activities, time of eating); ➄ postoperative transfer to ICU.	N
65	The benefits of a low dose complex carbohydrate/ citrulline electrolyte solution for preoperative carbohydrate loading: focus on glycemic variability	Kielhorn et al. (82)	The United States	Colorectal/small intestine surgery	83 cases (SIM group: 41; COM group: 42)	SIM group: consume commercial sports drinks ad libitum until hospital admission before surgery. (commercial sports drinks have an osmolality of 210–650 mOsm, containing 14–63 mg/dL simple carbohydrates and 0–3.6 mg/dL complex carbohydrates.)	COM group: consume 20 ounces of compound carbohydrate orally on the night before surgery, and consume 10 ounces orally 2 h before surgery. The preparation is maltodextrin/guanidine electrolyte solution manufactured by SOF health, containing 25 g maltodextrin and 3 g guanidine per 10 ounces (approximately 296 mL).	1. Main outcomes: ➀ perioperative blood glucose levels (monitored before surgery and within 1–3 days after surgery, with hyperglycemia defined as ≥140 mg/dL); ➁ blood glucose variability (number of hyperglycemic episodes/total days of hospitalization); 2. Secondary outcomes: ➀ complications 30 days after surgery (classified according to Clavien-Dindo classification); ➁ length of hospital stay; ➂ frequency of hyperglycemic episodes	Y
66	Effects of preoperative carbohydrate-rich drinks on immediate postoperative outcomes in total knee arthroplasty: a randomized controlled trial	Kadado et al. (83)	The United States	Unilateral total knee arthroplasty	153 cases (intervention group: 50; placebo group: 51;control group: 52)	Intervention group: drink 800 mL of Nutricia preOp orally on the night before surgery; drink 400 mL orally 3 h before surgery.	Placebo group: drink 800 mL of non-caloric beverage orally on the night before surgery; drink 400 mL orally 3 h before surgery. Control group: fast after midnight before surgery.	1. Main outcome: incidence of postoperative nausea and vomiting (PONV) (at three time points: 0–4 h, 4–12 h, and 12–24 h); 2. Secondary outcomes: ➀ length of hospital stay; ➁ opioid consumption (morphine milligram equivalent MME); ➂ VAS pain score (0–10 points); ➃ serum glucose level; ➄ postoperative fluid requirement; ➅ 90-days adverse events (emergency visits, readmissions, pulmonary embolism, etc.); ➆ patient-reported outcomes (KOOS JR, PROMIS scores).	N
67	A randomized controlled study of preoperative oral carbohydrate loading versus fasting in patients undergoing colorectal surgery	Rizvanović et al. (80)	Bosnia and Herzegovina	Open abdominal colorectal cancer surgery	50 cases (intervention group: 25; control group: 25)	Drink 400 mL of 12.5% maltodextrin orally on the evening before surgery; drink 200 mL orally 2 h before surgery.	Fast for 8 h before surgery.	1. Metabolic and inflammatory indicators: insulin resistance (HOMA-IR), insulin sensitivity (HOMA-ISI), Glasgow Prognostic Score (GPS), IL-6, CRP, albumin, were measured at T1 (6 o’clock on the surgery day), T2 (6 h after surgery), T3 (6 o’clock on the first day after surgery), and T4 (6 o’clock on the second day after surgery); 2. Subjective comfort: VAS scores for symptoms such as thirst, hunger, and dry mouth (0–10 points), were measured before induction of surgery and at 0–4/4–8/8–12/12–24 h after surgery; 3. Surgical clinical outcomes: recovery of gastrointestinal function (time of resumption of bowel sounds, time of first defecation/deflation, time of first eating), time of independent walking, time of postoperative discharge; 4. Others: incidence of nausea and vomiting, use of antiemetic drugs/analgesic drugs.	N
68	Effects of preoperative oral carbohydrate loading on neutrophil/ lymphocyte ratio and postoperative complications following colorectal cancer surgery: a randomized controlled study	Rizvanović et al. (81)	Bosnia and Herzegovina	Open abdominal colorectal cancer surgery	60 cases (intervention group: 30; control group: 30)	Drink 400 mL of Nutricia orally on the evening before surgery; drink 200 mL orally 2 h before surgery.	Fast for 8 h before surgery.	1. Main outcome: neutrophil/lymphocyte ratio (NLR), ΔNLR (maximum NLR after surgery - NLR before surgery), measured at 6 points before surgery (baseline), and at 6 points each on the 1st, 3rd, and 5th days after surgery; 2. Secondary outcome: Incidence and severity of complications within 30 days after surgery (classified according to Clavien-Dindo classification), including mild complications (grades I–II) and severe complications (grades III–V); 3. Others: length of hospital stay, intraoperative blood loss, blood transfusion status, etc.	N
69	The use of a pre-operative carbohydrate drink in patients with diabetes mellitus: a prospective, non-inferiority, cohort study	Laffin et al. (78)	Canada	Scheduled surgeries (including surgeries in cardiac surgery, neurosurgery, urology, general surgery and other departments)	106 cases (intervention group: 53; control group: 53)	Consume 500 mL of Ocean Spray or Minute Maid respectively 1 h before bedtime and 3 h before surgery. (Ocean Spray consists of natural fructose and added sugar, containing 12 g of carbohydrates per 100 mL, with a total of 60 g of carbohydrates per 500 mL serving.)	Fast for 8 h before surgery.	1. Primary outcome: preoperative blood glucose concentration (non-inferiority threshold: difference between groups > 2 mmol/L); 2. Secondary outcomes: ➀ preoperative incidence of hyperglycemia (blood glucose ≥ 11.1 mmol/L); ➁ length of hospital stay; ➂ preoperative insulin infusion rate; ➃ surgery cancellation rate; ➄ postoperative pneumonia incidence; ➅ 30-days mortality rate	N
70	Simple versus complex preoperative carbohydrate drink to preserve perioperative insulin sensitivity in laparoscopic colectomy a randomized controlled trial	Karimian et al. (79)	Canada	Laparoscopic colectomy	29 cases (simple CHO group: 15; complex CHO group: 14)	Simple CHO group: drink 400 mL of Minute Maid orally 2 h before surgery.	Complex CHO group: drink 400 mL of compound carbohydrate beverage orally 2 h before surgery. (40 g maltodextrin + 10 g fructose, with a osmotic pressure of 207 mOsm/kg and a pH of 4.5)	1. Primary outcome: intraoperative insulin sensitivity (measured by the hyperinsulinemic - normal glucose clamp method, with *M*-value < 5.5 mg/kg/min indicating insulin resistance); 2. Secondary outcomes: ➀ postoperative insulin resistance (HOMA2-IR), insulin sensitivity (HOMA2-% S), measured before and 1–3 days after surgery; ➁ fasting blood glucose (FBG), C-reactive protein (CRP); ➂ gastric residual volume; ➃ grip strength, health status VAS score; ➄ discharge preparation time, hospital stay; ➅ 30-days complications (classified according to Clavien-Dindo grading).	N
71	Influence of pre-operative oral carbohydrate loading vs. standard fasting on tumor proliferation and clinical outcome in breast cancer patients– a randomized trial	Lende et al. (84)	Norway	Breast cancer-related surgery	61 cases (intervention group: 26; control group: 35)	Drink 400 mL of Nutricia orally at 18:00 on the day before surgery and again 2–4 h before surgery.	Fast for 8 h before surgery.	1. Main outcome: tumor proliferation (measured by mitotic index MAI); 2. Secondary outcomes: ➀ serum indicators (insulin, insulin C-peptide, glucose, IGF-1, IGFBP3); ➁ patient comfort (pain, nausea, etc. within 1–7 days after surgery, 4-point Likert scale); ➂ long-term clinical outcomes (disease-free survival RFS, breast cancer-specific survival BCSS, overall survival OS); ➃ hormone receptor status (ER, PR, HER2).	N
72	Metabolic consequences of perioperative oral carbohydrates in breast cancer patients - an explorative study	Lende et al. (85)	Norway	Breast cancer-related surgery	60 cases (intervention group: 25; control group: 35)	Drink 400 mL of Nutricia orally at 18:00 on the day before surgery and again 2–4 h before surgery.	Fast for 8 h before surgery.	1. Main outcome: metabolic indicators (serum 28 metabolites: lactic acid, pyruvate, ketone bodies, etc.; tumor tissue 20 metabolites: glutathione, glutamate, etc.); 2. Secondary outcomes: ➀ proliferation indicators (MAI, Ki67, PPH3); ➁ survival outcomes (disease-free survival RFS, breast cancer-specific survival BCSS, overall survival OS); ➂ metabolic pathway analysis (IPA and MSEA).	N
73	The effect of preoperative oral carbohydrate or oral rehydration solution on postoperative quality of recovery: a randomized, controlled clinical trial	Asakura et al. (86)	Japan	Minimally invasive surgeries on the body surface (including urology, gynecology, plastic surgery, otolaryngology, orthopedics, and general surgery)	134 cases (carbohydrate group: 46; rehydration salt group: 43; control group: 45)	Carbohydrate group: drink 250 mL of Arginaid Water orally from 06:00 to 06:30 on the morning before surgery. Rehydration salt group: drink 1,000 mL of OS-1 orally from 20:00 on the night before surgery until 2 h prior to surgery.	Fast after midnight before surgery.	1. Primary outcome: quality of recovery 24 h after surgery [QoR-40 score, including 5 dimensions such as emotional state and physical comfort, ranging from 200 (worst) to 40 (best)]; 2. Secondary outcomes: ➀ use of vasopressors (ephedrine/phenylephrine) during surgery; ➁ changes in body temperature during surgery (from baseline to the maximum drop during surgery, and the change at the end of surgery); ➂ incidence of postoperative nausea and vomiting (PONV).	N
74	Effects of oral carbohydrate with amino acid solution on the metabolic status of patients in the preoperative period: a randomized, prospective clinical trial	Tsutsumi et al. (87)	Japan	Scheduled minor surgeries (including tubal and ovarian removal surgery, partial breast removal surgery, endoscopic sinus surgery, skin surgery, arthroscopic surgery, etc.)	24 cases (intervention group: 12; control group: 12)	Drink 500 mL of Arginaid Water^®^ orally 2 h before surgery.	Fast from 21:00 on the day before surgery.	1. Main outcomes: ➀ biochemical indicators (serum free fatty acids FFA, total ketones, blood glucose, insulin, etc.); ➁ metabolic indicators (respiratory quotient RQ, energy expenditure EE); 2. Secondary outcomes: ➀ subjective comfort (VAS score: anxiety, hunger, thirst, etc., 0 points indicate no discomfort - 100 points indicate the most severe); ➁ mental health score (PHQ-9 score, assessing depression-related symptoms)	N
75	Preoperative fasting abbreviation with whey protein reduces the occurrence of postoperative complications in patients with head and neck cancer: a randomized clinical trial	de Carvalho et al. (88)	Brazil	Head and neck cancer elective surgery	49 cases (intervention group: 26; control group: 23)	Drink 200 mL of the mixed solution 4 h before surgery, containing 7 g whey protein and 25 g maltodextrin.	Avoid solid food 8 h before surgery; drink 200 mL of NUTRI Dextrin Nutrimed orally 4 h before surgery.	1. Primary outcome: incidence of postoperative complications (determined according to the Clavien-Dindo classification criteria); 2. Secondary outcomes: ➀ nutritional status (PG-SGA score and classification, BMI); ➁ biochemical indicators (blood glucose, insulin, C-reactive protein, cortisol, HOMA-IR, QUICKI); ➂ surgery-related indicators (operation duration: ≤4 h/>4 h); ➃ postoperative recovery (early activity status); ➄ hospital stay; ➅ 30-days mortality rate.	N
76	Reducing pre-operative fasting while preserving operating room scheduling flexibility: feasibility and impact on patient discomfort	De Jonghe et al. (89)	France	Abdominal surgeries: weight loss, colorectal, biliary tract, etc.; gynecological surgeries: uterine appendages, breast, etc.; orthopedic surgeries: hip and knee surgeries, internal fixation removal, etc.	393 cases (intervention group: 199; control group: 194)	For morning surgery (08:30–12:59), drink 400 mL of Clinutren-Preload orally 3 h before surgery; for afternoon surgery (13:00–16:59), drink 400 mL of Clinutren-Preload orally at 10:00.	Fast after midnight before surgery.	1. Primary outcome: feasibility (delivery rate) of preoperative oral carbohydrate administration; 2. Secondary outcomes: ➀ preoperative fasting duration; ➁ perioperative comfort (thirst, hunger, anxiety, weakness, measured before surgery and upon return to the ward using a 0–10 numerical rating scale)	N
77	Effect of pre-operative oral carbohydrate loading on recovery after day-case cholecystectomy: a randomized controlled trial	Helminen et al. (90)	Finland	Laparoscopic cholecystectomy	113 cases (intervention group: 57; control group: 56)	Drink 200 mL of Providextra^®^ orally 2–3 h before surgery.	Fast after midnight before surgery.	1. Main outcome: 6 types of perioperative discomfort VAS scores (ranging from 0 to 100 points), including thirst, hunger, dry mouth, fatigue, nausea, and pain, were measured at admission, before induction, 2 h/4 h after surgery, and at discharge; 2. Secondary outcomes: ➀ use of analgesics/antiemetics; ➁ postoperative water intake, food intake, and first activity time; ➂ discharge time; ➃ readmission rate.	N
78	The effects of honey solution on postoperative stress, gastric motility, and patient comfort: a randomized controlled trial	Tarčuković et al. (95)	Croatia	Laparoscopic cholecystectomy	55 cases (HS group: 20; CCB group: 25)	HS group: drink 800 mL of honey solution orally on the night before surgery; drink 400 mL orally 2 h before surgery. (The 800 mL solution contains 100 g carbohydrates and 400 kcal, and the 400 mL solution contains 50 g carbohydrates and 200 kcal)	CCB group: drink 800 mL of Preop orally on the night before surgery; drink 400 mL orally 2 h before surgery.	1. Main outcomes: ➀ stress and inflammation indicators (cortisol, IL-6, measured at multiple time points); ➁ gastric motility (acetaminophen absorption test, blood drug concentration measured at 15/30/60/120/180 min after administration); 2. Secondary outcomes: patient comfort VAS score (measured on the first postoperative day, including thirst, nausea, pain, vomiting, appetite, overall well-being); 3. Others: blood glucose level (measured before bedtime, on the day of surgery, and on the first postoperative day).	N
79	The effects of preoperative oral carbohydrate drinks on energy intake and postoperative complications after hip fracture surgery: a pilot study	Loodin et al. (96)	Sweden	Hip fracture-related surgery	109 cases (intervention group: 59; control group: 50)	Drink 200 mL of carbohydrate solution orally on the evening before surgery and 2 h before surgery. (Prepare by mixing 50 g powder with 200 mL water; each 50 g portion contains 47.5 g carbohydrates and 190 kcal.)	Avoid solid food for 6 h before surgery and refrain from drinking water for 2 h before surgery.	1. Main outcomes: ➀ rate of energy intake reaching the target (calculated based on hospital guidelines, combined with age, BMI, and activity level); ➁ incidence of postoperative complications (pressure ulcers, wound infections, urinary tract infections, pneumonia, etc.); 2. Secondary outcomes: length of hospital stay, ASA classification, cognitive status (screened by SPMSQ).	N
80	Preoperative oral carbohydrate load versus placebo in major elective abdominal surgery (PROCY): a randomized, placebo-controlled, multicenter, phase III trial	Gianotti et al. (91)	Italy	Scheduled major abdominal surgeries (including surgeries on the stomach, colon and rectum, liver, kidney, prostate, uterus/ovaries, etc.)	662 cases (intervention group: 331; control group: 331)	Drink 800 mL of PeriOp orally beginning at 20:00 on the night before surgery and discontinue intake 2 h prior to surgery.	Drink 800 mL of clear water orally. Start drinking at 8 o’clock the night before surgery and stop drinking 2 h before surgery.	1. Main outcome: postoperative infections (wound infection, organ/cavity infection, urinary tract infection, pneumonia, sepsis/septic shock); 2. Secondary outcomes: ➀ number of patients with hyperglycemia (blood glucose >110–140 mg/dL, >140–180 mg/dL); ➁ insulin usage during and after the operation; ➂ proportion and duration of empirical antibiotic use; ➃ incidence and severity of postoperative complications (Dindo-Clavien classification); ➄ rate of reoperation, ICU stay, postoperative hospital stay; 3. Safety indicators: residual gastric volume >100 mL, aspiration of lung, nausea and vomiting, etc.	Y
81	Efficacy of preoperative oral glucose on blood glucose response and neutrophil–lymphocyte ratio in patient undergoing brain tumor resection: randomized controlled trial study	Senapathi et al. (94)	Indonesia	Craniotomy for tumor removal	68 cases (intervention group: 34; control group: 34)	Drink 400 mL of 12.5% maltodextrin orally 2 h before the operation (50 g/400 mL, 0.5 calories/mL).	Drink 400 mL of clear water orally 2 h before the operation.	1. Main outcomes: ➀ blood glucose levels (measurement time points: 1 h before surgery, before anesthesia induction, 6 h after surgery, 24 h after surgery); ➁ neutrophil-to-lymphocyte ratio (NLR, the same as blood glucose measurement time points); 2. Safety indicators: intraoperative bleeding, operation duration, postoperative complications.	N
82	Preoperative maltodextrin solution intake improves patient comfort in gynecological laparoscopic surgery: a randomized controlled trial	Van Loi et al. (92).	Vietnam	Gynecological laparoscopic surgery	70 cases (intervention group: 35; control group: 35)	Drink 600 mL of 15% maltodextrin orally on the evening before surgery. Drink 300 mL orally 2 h before surgery.	Fast for 8 h before surgery.	1. Main outcome: perioperative comfort (VAS score, 0–10 points), including scores for hunger, thirst, and fatigue before anesthesia and 2 h after surgery; 2. Secondary outcomes: ➀ baseline characteristics (age, BMI, ASA classification, type of surgery/duration); ➁ volume of intravenous fluid administration during surgery, amount of ephedrine used; ➂ preoperative gastric residual volume (assessed by ultrasound).	N

**TABLE 2:** Product name and ingredient list.

Product name	Manufacturer and composition	Product name	Manufacturer and composition
Su Qian	Product name, manufacturer: Jiangsu Zhengda Fenghai Pharmaceutical Co., Ltd.; concentration: 12.6%: maltodextrin fructose beverage; energy: 50 kcal/100 mL; osmotic pressure: 290 mOsm/kg; pH: 5.0; specification: 200 mL/Bottle	PreopQ/PeriOp	Product name: Nutricia preOp; production company: Nutricia of the Netherlands; components: carbohydrates: 12.6 g; energy: 50 kcal (215 kJ); osmotic pressure: 240 mOsmol/L
Shuneng	Manufacturer: Yichang Renfu Pharmaceutical Co., Ltd.; ingredients: carbohydrates, sodium, vitamins B1/B6/B12, zinc, taurine; specification: 300 mL per dose.	NO-NPO	Product name: NO-NPO Manufacturer: Daesang WelLife, Seoul, Republic of Korea Composition: 12.8% carbohydrates, 0.5 kcal/mL, osmolality 290 mOsm/kg
Outfast	Product name: Outfast; manufacturer: Humanwell Health Care; ingredients: 10 g maltodextrin, 2.4 g fructose, 1.7 g glucose, and 1.2 g taurine per 100 mL; energy: 241 kJ per 100 mL; specification: 355 mL per bottle.	Resource^®^ Breeze	Product name: Resource^®^ Breeze Manufacturer address: Petaling Jaya, Selangor, Malaysia Concentration/composition: Per 237 mL: 250 kcal, 9 g whey protein, 48 g carbohydrates Proportion: 14% whey protein, 86% carbohydrates, 0% fat, lactose-free Appearance: clear, amber-colored, fruit-flavored Osmolality: 921 mOsm/kg
EHR	Product name: Bangshidi; manufacturer: Guangdong Bangshidi Medical Food Co., Ltd; ingredients: enzymatically broken rice flour (EHR, water-soluble small molecule carbohydrates); specification: 20 g per package; each 100 g contains 93 g of carbohydrates, 2 g of dietary fiber, 200 mg of sodium, no protein or fat	Minute Maid	Product name: Minute Maid 100% Pure Apple Juice Manufacturer: The Minute Maid Company, a subsidiary of The Coca-Cola Company Ingredients: concentrated apple juice, water; contains natural fructose and glucose, no added sugar Nutritional composition: 12 g of carbohydrates per 100 mL liquid; 60 g of carbohydrates per 500 mL serving
Dongze Sutang	Product name: Dongze Sutang; production company: Jiangsu Dongze Special Medical Food Co., LTD. Ingredients: maltodextrin, dietary glucose, crystalline fructose (96 g carbohydrate per 100 g); The concentration was 12.48 g/100 mL for two doses.	Arginaid Water™	Product name: Arginaid Water Manufacturer: Nestlé Health Science Composition and specification: contains 18% carbohydrates and 2% L-arginine; energy density 0.8 kcal/mL; osmolality 545 mOsm/L.
Mix carbohydrate solution	Each 100 mL contains glucose 0.2 g, fructose 1.3 g, maltose 0.7 g, polysaccharide 10 g, sodium, potassium, chlorine and other trace elements, pH4.9	OS-1™	Product name: OS-1 Manufacturer: Otsuka Pharmaceutical Factory, Inc. Composition: contains 2.5% carbohydrates including 1.8% glucose; energy density 0.1 kcal/mL; osmolality 270 mOsm/L.
Compound carbohydrate nutritional preparation	Name of the product; company: Department of Nutrition, West China Hospital, Sichuan University; concentration: the preoperative preparation contained 50 g of carbohydrate per 200 mL	Fresubin Protein Powder	Product name: Fresubin Protein Powder Manufacturer: Fresenius Kabi Composition: mainly whey protein; nearly free of carbohydrates and fat, high in protein concentration.
Junbeian	Product name: Junbeian; production company: Guangdong Junyue Nutrition Medical Co., LTD.; concentration: 12.5% (special medical use carbohydrate component formula food, the main ingredient is maltodextrin, Chinese food note: TY20210007)	NUTRI Dextrin Nutrimed	Product name: NUTRI Dextrin Nutrimed Manufacturer: Danone Nutrimed Composition: main component is maltodextrin, used as a pure carbohydrate supplement.
Fuan	Product name: Fuan; production company: Hainan Donglian Changfu Pharmaceutical Co., LTD. concentration: 12.6% (carbohydrate), energy 214.2 kJ/100 mL, osmolality 280 mOsm/kg	Clinutren-Preload	Product name: Clinutren-Preload Manufacturer: Nestlé Health Science Composition and specification: each sachet contains 47.5 g of neutral-flavor carbohydrates. When diluted with 400 mL of water, the osmolality is 135 mOsm/kg with an energy content of 190 kcal.
Mizone	Product name: Mizone vitamin drink; production company: Danone Group; concentration: 9.8% (9.8 g carbohydrate per 100 mL)	Providextra^®^	Product name: Providextra^®^ Manufacturer: Fresenius Kabi, Germany Composition and specification: each 200 mL contains 300 kcal energy, 67 g carbohydrates, and 8 g protein.

### Geographic and temporal distribution of research

**FIGURE 2:**
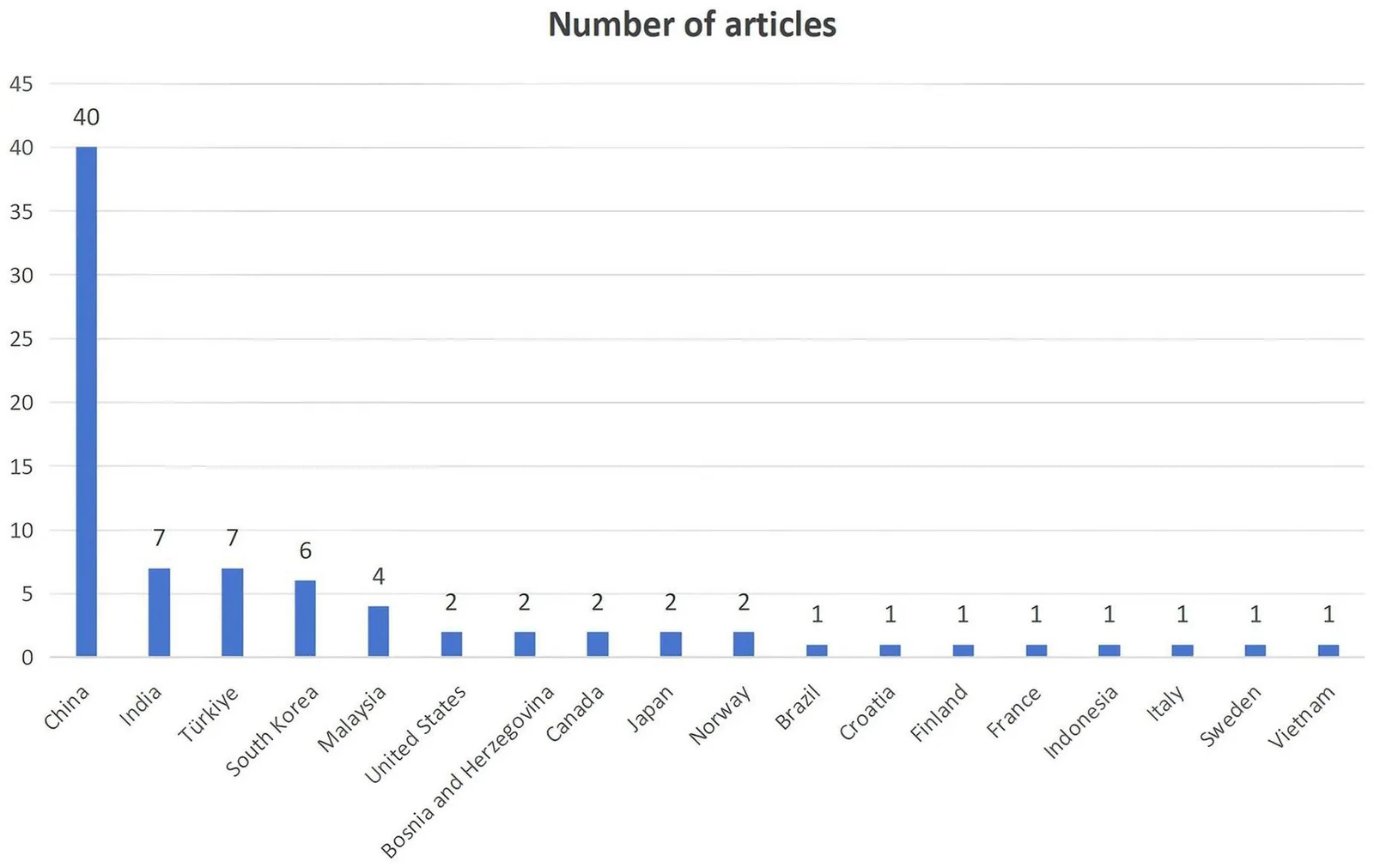
Number of included studies by country or region. China accounted for the largest proportion (40 studies), followed by India and Türkiye (7 each), South Korea (6), Malaysia (4), and the remaining countries with one or two studies each.

The included studies covered 18 countries and regions (Figure 2). Among these records, 40 studies were conducted in China (14–53), accounting for 48.8% of all included literature, making China the largest contributor of relevant research in the sample. India (54–60) and Turkey (61–67) each produced 7 studies, both constituting an identical proportion of 8.5%. South Korea contributed 6 studies (68–73), representing 7.3% of the total. All remaining countries individually contributed less than 5% of included studies, including Malaysia [4 studies (74–77)], Canada [2 studies (78, 79)], Bosnia and Herzegovina [2 studies (80, 81)], the United States [2 studies (82, 83)], Norway [2 studies (84, 85)], and Japan [2 studies (86, 87)]. Brazil (88), France (89), Finland (90), and other nations each supplied one study.

**FIGURE 3:**
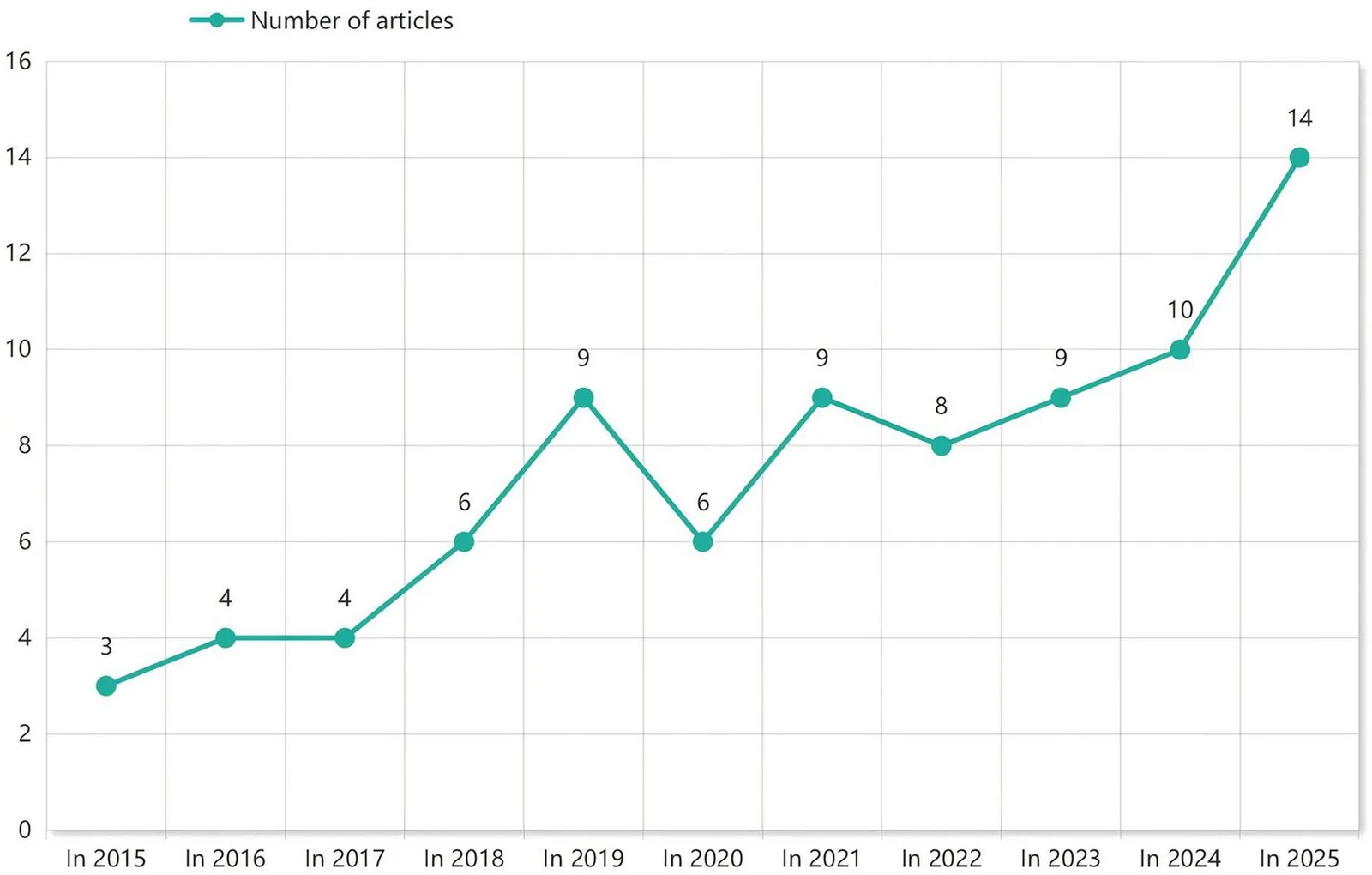
Number of included studies by year of publication (2015–2025). The annual number of publications showed an overall upward trend, increasing from 3 in 2015 to a peak of 14 in 2025, with minor fluctuations.

The studies were published between 2015 and 2025. A total of 26 studies were published before 2020 (12, 14, 19, 20, 22, 29, 41, 54, 61–65, 68–70, 78, 80, 82, 84–87, 89–91), accounting for 31.7% of the total. From 2020 to 2025, 56 studies were published (15–18, 21, 23–28, 30–32, 34–40, 42–53, 55–60, 66, 67, 71–77, 79, 81, 83, 88, 92–95), representing 68.3% of the total, as shown in Figure 3.

### Study design characteristics

The study designs of the included studies fall into two categories, with randomized controlled trials (RCTs) as the core and non-randomized controlled trials (non-RCTs) as a supplement, where the evidence levels are appropriately aligned with the research objectives. Specifically, there were 76 RCTs, accounting for 92.7% of the total The 6 non-RCT studies (43, 48, 49, 51, 52, 78), representing 7.3% of the total, included 3 prospective cohort studies (43, 49, 78), 1 retrospective cohort study (51), 1 retrospective study (48), and 1 cross-sectional study (52).

In terms of the scale of research implementation, single-center studies occupy an absolute dominant position, while the number of multi-center studies remains limited. A total of 74 single-center studies (90.2% of the total) were identified, which were mostly conducted by individual hospitals or departments. There were 8 multi-center studies (19, 30, 32, 35, 37, 82, 90, 91), accounting for 9.8% of the total, all of which were carried out in regions with concentrated medical resources, including 5 from China (19, 30, 32, 35, 37), 1 from Italy (91), 1 from Finland (90), and 1 from the United States (82).

Blinding was only applicable to randomized controlled trials (RCTs); non-RCTs were not blinded due to their inherent design characteristics. Among the 76 included RCTs, 40 studies (52.6%) adopted open-label or unblinded designs (14, 17–21, 23–30, 33, 38, 44, 47, 53, 55–59, 61–65, 67, 68, 71–74, 76, 82, 89, 95, 96); 25 studies (32.9%) used single-blind designs (15, 16, 32, 34–36, 39, 41, 42, 46, 50, 60, 66, 69, 75, 80, 81, 84–88, 90–92), where blinding was typically implemented for outcome assessors or data analysts, while participants were fully aware of their group allocation. Eleven studies (14.5%) employed double-blind designs (22, 31, 37, 40, 45, 54, 70, 77, 79, 83, 94), which were only achievable via “placebo beverages with identical appearance and taste” (e.g., calorie-free flavored water with a formulation similar to the carbohydrate drink).

### Characteristics of the study population

All included study participants were elective surgery patients aged 18 years or older, with no minors or emergency surgery patients enrolled. The study population was highly aligned with specific surgical types: predominantly patients undergoing orthopedic procedures (hip and knee arthroplasty), gastrointestinal surgery (laparoscopic cholecystectomy, radical resection for gastrointestinal carcinoma), gynecological surgery (laparoscopic total hysterectomy, cervical cancer surgery), thoracic surgery (video-assisted thoracoscopic lobectomy), and endoscopic procedures [endoscopic submucosal dissection (ESD) for early gastric cancer]. A small number of patients undergoing radical thyroidectomy and lumbar spine surgery were also included, all of whom were eligible for preoperative oral carbohydrate intervention based on clinical indications.

Among the included studies, several studies enrolled adults across the full age spectrum (18–60 years or 18–80 years), while 13 studies specifically targeted older adults (≥60 or ≥65 years) (15–17, 21, 22, 28, 29, 34, 36, 40, 45, 50, 76) accounting for 15.9% of all included studies. Among these geriatric-focused studies, eight involved orthopedic procedures (17, 21, 22, 28, 29, 40, 50, 76)–predominantly unilateral or bilateral total knee arthroplasty and total hip arthroplasty–while the remaining five covered video-assisted thoracoscopic lobectomy (15), open abdominal gastrointestinal surgery (16, 34), open radical prostatectomy (45), and diagnostic or therapeutic upper gastrointestinal endoscopy (36). All studies restricted inclusion to patients classified as American Society of Anesthesiologists (ASA) physical status class I–III. The majority enrolled low- to moderate-risk patients (ASA I–II), whereas a limited number selectively included stable ASA III patients–defined as those with well-controlled comorbidities–such as in studies on early gastric cancer treated by endoscopic submucosal dissection (ESD) or surgical management of hip fractures in older adults.

The inclusion and exclusion criteria were clearly safety-oriented. Regarding diabetes–a key comorbidity in preoperative carbohydrate intervention–only three studies specifically included such patients (49, 51, 78) (3.7%), involving unilateral joint replacement, coronary artery bypass grafting, and elective multi-disciplinary surgeries across cardiac, neurosurgical, urological, and general surgical fields, all with the restriction of “stable blood glucose control.” Most other studies excluded patients with diabetes, impaired glucose tolerance, severe hypertension, cirrhosis, chronic kidney disease, and similar underlying conditions. All studies excluded individuals at high risk of aspiration, such as those with gastric emptying disorders (gastroparesis, pyloric obstruction) or gastroesophageal reflux. Some additionally excluded patients with abnormal BMI (<18.5 kg/m^2^ or >30 kg/m^2^) or malnutrition (serum albumin <35 g/L), aiming primarily to avoid potential safety risks and the impact of absorption abnormalities on outcomes. Commonly applied exclusion criteria also included severe heart, liver, or kidney disease; acute infection within 24 h before surgery; cognitive or communication impairments; carbohydrate allergy; and prior gastrointestinal surgery. Depending on the surgical type, some studies further excluded pregnant women and advanced cancer patients.

### Distribution of surgical procedures

The 82 included studies spanned 14 surgical specialties. Due to overlapping classification criteria–where some studies were coded at both the subspecialty level (e.g., “orthopedic surgery: hip/knee arthroplasty”) and the broader surgical category level (e.g., “orthopedic surgery” alone)–the sum of specialty-specific counts exceeds the total number of studies. Therefore, all reported proportions are calculated relative to the denominator of 82 studies. To ensure conceptual clarity and avoid double-counting in interpretation, the distribution is presented based on the *primary surgical specialty* assigned to each study–that is, the specialty most directly aligned with the index procedure and central to the study’s clinical rationale and patient population.

Gastrointestinal surgery was the most frequently represented specialty, accounting for 19 studies (19, 27, 30, 34, 35, 37, 38, 44, 49, 60, 71, 75, 77, 79–82, 89, 91) (23.2%; 19/82). These comprised predominantly elective procedures: laparoscopic radical resection for gastric or colorectal cancer (*n* = 10), open abdominal gastrointestinal surgery (*n* = 6), and endoscopic polypectomy (*n* = 3). Orthopedic surgery ranked second, with 16 studies (16, 17, 21, 22, 28, 29, 40, 50, 57, 61, 72, 76, 83, 86, 89, 96) (19.5%; 16/82). The majority involved major joint arthroplasty–unilateral or bilateral total knee arthroplasty (*n* = 8) and total hip arthroplasty (*n* = 5)–alongside surgical management of hip fractures (e.g., intramedullary nailing or dynamic hip screw fixation; *n* = 3).

Hepatobiliary surgery was represented by 18 studies (14, 23–25, 31, 32, 39, 46, 52, 54, 55, 59, 65, 66, 70, 90, 91, 95) (22.0%; 18/82). Laparoscopic cholecystectomy constituted the predominant procedure (*n* = 12), followed by endoscopic retrograde cholangiopancreatography (ERCP; *n* = 3) and open hepatobiliary surgery (*n* = 3)–all elective biliary tract interventions. Gynecologic surgery included 10 studies (26, 43, 49, 52, 58, 74, 86, 89, 91, 92) (12.2%; 10/82), primarily comprising laparoscopic total hysterectomy (*n* = 6), radical hysterectomy for cervical cancer (*n* = 2), and tubal procedures (*n* = 2). All were elective pelvic surgeries (either minimally invasive or open), with postoperative recovery shown to be sensitive to preoperative nutritional status–particularly in terms of muscle mass preservation and immune function.

Endoscopic procedures were represented by 7 studies (36, 41, 51, 53, 73, 75, 87) (8.5%; 7/82), including 4 cases of endoscopic submucosal dissection (ESD) for early gastric cancer and 3 colonoscopic therapeutic interventions–all minimally invasive endoscopic operations. Urological surgery comprised 5 studies (30, 45, 78, 86, 91) (6.1%; 5/82), primarily transurethral resection of the prostate (TURP); cardiovascular surgery included 4 studies (42, 63, 68, 78) (4.9%; 4/82), mainly coronary artery bypass grafting (CABG); thyroid surgery involved 3 studies (18, 64, 69) (3.7%; 3/82), predominantly radical thyroidectomy for thyroid cancer. Head and neck surgery accounted for 4 studies (47, 48, 86, 88) (4.9%; 4/82), breast surgery 2 studies (84, 85) (2.4%; 2/82), spinal surgery 3 studies (20, 62, 67) (3.7%; 3/82), and neurosurgery 2 studies (33, 94) (2.4%; 2/82).

Three specific scenarios requiring special note regarding double counting are as follows: First, five studies labeled as “elective major open surgery” include gastrointestinal and gynecological surgeries; these studies are counted only once under the “core specialty” (e.g., general surgery), to avoid overestimating their proportion. Second, four studies categorized as “elective minor surgery” cover procedures such as tubal surgery, mastectomy, and endoscopic surgery; they are assigned to the “most relevant specialty” (e.g., gynecology or breast surgery) and counted once. Third, two studies labeled as “multidisciplinary elective surgery” involve multiple specialties including gastroenterology, hepatobiliary, and gynecology; these are also counted once based on the “specialty corresponding to the primary study population.” Although this classification results in a total exceeding 82 studies, the percentage calculations are always based on the total number of publications, preserving the core finding that preoperative carbohydrate interventions primarily target minimally invasive, elective, and common surgical specialties.

### Intervention and control protocol

All 82 studies comprehensively documented carbohydrate type, dosage, and administration timing, with no missing core information. Maltodextrin-based formulations dominated as the primary carbohydrate type, accounting for 31 studies (20, 21, 23, 25, 26, 28–34, 37, 42, 43, 45–49, 51, 53, 57, 59, 60, 66, 75, 80, 92, 94) (37.8%). These included pure maltodextrin solutions (12.5%–15% concentration), maltodextrin-containing compound preparations (supplemented with fructose, glucose, or electrolytes such as sodium, potassium, and zinc), and commercial specialized beverages with maltodextrin (e.g., Shuneng, Suqian, Nutricia preOp™). Glucose solutions were utilized in 10 studies (15, 18, 22, 24, 27, 35, 36, 38, 56, 65) (12.2%), predominantly at concentrations of 5% and 10%. Other carbohydrate types were reported in 7 studies (16, 39, 77–79, 82, 95) (8.5%), including honey solutions, enzymatically hydrolyzed rice powder, and cocktail beverages. These formulations are largely region-specific–for instance, Kelulut honey from Malaysia (77), Ocean Spray from Canada (82), and M脉动 (M-Pulse) from China (16)–and primarily consist of natural carbohydrates (fructose, glucose), though they have been applied only in a limited number of studies. Among the 82 included studies, administration timing was predominantly a single dose 2–3 h before surgery; 41 studies adopted a dual oral regimen combining an evening dose before surgery and a dose 2 h preoperativel (18, 24, 27, 31, 37, 38, 40–42, 45, 48, 51–54, 58–65, 68, 69, 71, 74, 76–78, 80–86, 91, 92, 95, 96), with a total volume of 800–1200 mL. The dual regimen showed no fixed association with surgical type. Formulations were predominantly commercial products from brands such as Yichang宜昌人福 (Yichang Humanwell), Jiangsu正大丰海 (Jiangsu Zhengda Fenghai), Nutricia, and Abbott, with clear ingredient labeling and standardized concentrations (primarily 12%–15%). Six studies (7.3%) used hospital-prepared formulations, e.g., the compound carbohydrate preparation from the Nutrition Department of West China Hospital, Sichuan University, and 14.2% clear carbohydrate solutions prepared by hospital pharmacies (14, 19, 40, 41, 44, 96). These hospital-prepared formulations were often customized with ingredients (e.g., trace elements, dietary fiber) based on surgical type to meet the metabolic needs of specific populations (e.g., obese or elderly patients).

There are three main control schemes: first, blank control, in which the control group follows a traditional fasting protocol without any oral fluid intervention; only a few studies administer intravenous glucose solution when patients experience hypoglycemia or other special conditions. This type of control serves as the baseline for evaluating preoperative carbohydrate interventions. Second, placebo control, where the control group ingests an equal volume of non-carbohydrate liquid–such as warm water, purified water, or flavored water (matched in appearance and taste to carbohydrate beverages)–and some studies use oral rehydration salts (containing minimal carbohydrates, thus negligible intervention effect). Third, crossover comparison, in which no separate blank or placebo control is used; instead, different experimental groups are compared against each other based on varying intervention parameters, such as different dosages (e.g., 100 mL vs. 200 mL carbohydrate solution), different carbohydrate types (e.g., simple vs. complex carbohydrates), or different administration frequencies (single vs. double doses).

### Outcome measures covered

The outcome measures focus on five core dimensions: “metabolism, comfort, inflammation, clinical recovery, and safety,” providing comprehensive and clearly structured coverage.

The coverage rate of metabolism-related indicators was 56.1%, serving as the core evaluation dimension across all studies and forming a testing system of “basic indicators + characteristic indicators”: Basic indicators included fasting blood glucose, serum insulin levels, and the homeostatic model assessment of insulin resistance (HOMA-IR). Measurement time points were concentrated at 1 h preoperatively, 2 h postoperatively, 24 h postoperatively, and 3 days postoperatively; in some studies involving cardiovascular surgery patients or diabetic patients, assessments were extended to 5 days postoperatively. Thirty-five studies reported postoperative HOMA-IR or related insulin-resistance indicators (16, 17, 19–24, 26, 27, 33, 37–39, 42, 46, 59, 61–66, 68, 69, 72, 77–80, 82, 84, 87, 88, 91). The direction of findings was generally favorable, with many studies reporting lower postoperative insulin resistance in carbohydrate groups; however, heterogeneity in surgical populations, formulations, dosing regimens and measurement time points precluded quantitative pooling in this scoping review.

The coverage rate of patient comfort indicators was 46.3%. Assessment tools were dominated by standardized scales, balancing subjectivity and comprehensiveness: The core assessment employed the Visual Analog Scale (VAS) to measure hunger, thirst, and anxiety (scored 0–10 or 0–100). Some studies supplemented this with the Self-Rating Anxiety Scale (SAS), Quality of Recovery-15/40 (QoR-15/QoR-40), and General Comfort Questionnaire (GCQ).

The coverage rate of inflammation and stress markers was 15.9%, focusing on core targets of surgical stress and inflammatory responses and forming a panel of “conventional inflammatory factors + stress hormones + novel inflammatory markers.” Conventional markers included interleukin-6 (IL-6), tumor necrosis factor-α (TNF-α), and C-reactive protein (CRP). Thirteen studies utilized these as outcome measures (20, 23, 38, 40, 42, 45, 48, 50, 59, 61, 62, 80, 95), with measurement time points predominantly at 24 h postoperatively (postoperative day 1) and 48 h postoperatively (postoperative day 2). Stress hormones encompassed serum cortisol and adrenocorticotropic hormone (ACTH), which were primarily applied in studies involving major orthopedic and cardiovascular surgical procedures.

The coverage rate of clinical recovery and prognostic indicators was 43.9%, exhibiting a clear gradient distribution based on follow-up intervals: Short-term prognosis (within 1 month postoperatively) indicators were the most extensively covered. Ten studies focused on mortality, unplanned readmission rates, and complication rates (e.g., infection, anastomotic leakage, thrombosis) within 1 month postoperatively–these constitute the core content of current prognostic assessment (33, 35, 38, 40, 57, 78, 79, 81, 82, 88). In contrast, only one study investigated 1-year postoperative mortality (40), and no studies addressed key long-term outcomes such as functional recovery or quality of life changes between 3 and 12 months postoperatively.

The coverage rate of safety-related indicators was 24.4%, focusing on the safety boundaries of preoperative carbohydrate intervention with a core focus on “aspiration risk + metabolic safety”: Aspiration-related indicators included gastric residual volume (assessed via ultrasound or nasogastric tube aspiration), gastric antral cross-sectional area (CSA), and Perlas grading. No studies reported an increased risk of aspiration associated with preoperative carbohydrate intervention. Twenty-two of the included studies reported gastric residual volume after preoperative oral carbohydrate administration as one of their safety outcomes

## Discussion

### Research focus evolution: a multidimensional driven transformation from “utility validation” to “precision safety”

From the temporal distribution observed across the 82 included studies, trends in research on preoperative oral carbohydrate administration appear to shift from basic efficacy validation toward targeted precision and safety management. Such shifts may be linked to evolving clinical demands, technical improvements in perioperative monitoring, and the maturation of evidence-based ERAS practice. Since 2020, research priorities have shifted to defining safety thresholds and stratified patient management, largely driven by updated clinical testing tools. Gastric ultrasound has enabled quantitative safety evaluation: early aspiration risk assessments relied on subjective observation, while recent trials measure gastric antral cross-sectional area, gastric volume and emptying rate to establish population-specific safety benchmarks (30, 36, 77). High-precision glucose monitoring also captures dynamic glycemic variability, facilitating safer carbohydrate protocols for patients with diabetes.

Modern research additionally stresses contraindication screening and risk-stratified care. Integrated evidence suggests diabetic candidates should maintain preoperative blood glucose between 7.8 and 10 mmol/L before carbohydrate intake, whereas patients with gastroesophageal reflux or delayed gastric emptying are typically excluded (13). This shift from generalized universal administration to individualized, risk-adjusted practice aligns with the precision, patient-centered core of contemporary ERAS principles.

### Multidimensional heterogeneity of perioperative oral carbohydrate regimens: interactive drivers from surgical categories, patient populations and trial methodological designs

Observable discrepancies in oral carbohydrate delivery regimens can be identified across the included body of literature, and such inter-study variations may correlate with three mutually interactive dimensions: surgical procedure characteristics, baseline patient risk profiles, and the methodological arrangements of primary trials.

#### Surgery-specific regimen differentiation: regulating dosage and administration timing based on surgical trauma and perioperative fasting demands

Variations in carbohydrate volume, total carbohydrate load and dosing schedules appear to correspond closely with the magnitude of surgical trauma and standard perioperative fasting routines for distinct operations. Minimally invasive endoscopic and laparoscopic procedures tend to adopt single low-volume carbohydrate preparations, a pattern consistent with short preoperative fasting windows and mild intraoperative metabolic stress. For major open abdominal surgeries and large joint replacement orthopedic procedures, dual-dose regimens consisting of evening pre-operative intake combined with a second dose 2–3 h before incision are more frequently reported, a practice that may alleviate prolonged preoperative hunger induced by extended nil per os orders. By contrast, complex high-risk cardiac and neurosurgical interventions show inconsistent and limited standardized carbohydrate protocols across existing evidence, with no unified administration strategy widely adopted in current clinical trials. The adaptive adjustment of carbohydrate schemes according to surgical subtypes reflects pragmatic clinical balancing between intervention efficacy and perioperative operational feasibility.

#### Population-based stratified implementation divergence: differentiated application according to patient comorbidities, age and ASA risk grading

The general study population recruited within most primary investigations centers on low-risk elective patients classified as ASA I–II with intact gastrointestinal and glycemic function, for whom unified carbohydrate loading protocols are universally applied. Subgroup research targeting elderly surgical patients routinely modifies total carbohydrate loads and strengthens gastric volume safety monitoring via ultrasound assessment (1, 2, 12, 13). Patients complicated by diabetes mellitus, renal and hepatic dysfunction, or pathological delayed gastric emptying are largely excluded from routine carbohydrate intervention in most trial. Such differentiated inclusion and implementation standards generate substantial heterogeneity in real-world translational application, as one-size-fits-all protocols derived from low-risk cohorts cannot be directly extrapolated to multimorbid high-risk surgical groups.

#### Methodological heterogeneity across primary trials: impacts of randomization, blinding design and single/multicenter settings on intervention protocols

Methodological design differences among original trials further amplify regimen inconsistency. Randomized controlled trials constitute the dominant research type, yet open-label designs prevail in most published works, as sensory discrepancies between carbohydrate drinks and water-based control solutions create practical barriers to successful double-blinding. Single-center studies tend to adopt customized compound formulations prepared by local hospitals to match institutional supply conditions, while multicenter investigations predominantly select commercially standardized maltodextrin preparations to guarantee consistent intervention implementation across participating wards. Such trial-design driven differences in formula selection generate additional cross-study variation in carbohydrate concentration, osmolality and component proportions, which complicates direct comparative analysis of clinical outcomes across independent investigations.

### Heterogeneity in outcome evaluation systems: uneven distribution, divergent detection tools and distinct observation time windows of outcome indicators

Analysis of outcome measurement frameworks documented within the included literature reveals unbalanced evaluation priorities, inconsistent detection modalities and narrow postoperative observation intervals, forming a fragmented assessment system for preoperative oral carbohydrate interventions.

Metabolic indices and patient-reported subjective comfort metrics serve as core universal evaluation endpoints covered by nearly all relevant studies. Metabolic assessments primarily focus on perioperative blood glucose fluctuation and insulin resistance indicators, while subjective experience is quantified via standardized VAS scales for hunger, thirst and discomfort. In contrast, inflammatory stress markers, long-term postoperative functional recovery and chronic quality-of-life outcomes are rarely incorporated into trial evaluation plans, resulting in insufficient evidence describing the sustained clinical benefits of carbohydrate loading beyond the immediate perioperative period.

Even for identical safety and metabolic endpoints, divergent detection tools are adopted across trials. Objective aspiration risk assessment ranges from qualitative clinical observation to quantitative ultrasound measurement of gastric antral cross-sectional area and total gastric volume (1, 13, 17), without a unified gold-standard evaluation framework accepted globally. Glycemic monitoring also varies widely, from discrete single-point blood glucose testing to continuous dynamic glucose tracking that captures glucose variability, creating inconsistent reference benchmarks for judging glycemic safety during carbohydrate intervention (1, 13, 17).

Furthermore, outcome measurement time windows remain confined to the early postoperative 24–72 h in the vast majority of primary studies. Extended follow-up assessments addressing medium and long-term postoperative complications, rehabilitation speed and readmission events are seldom reported. This uniform reliance on short-term observation windows may originate from higher resource and time costs associated with prolonged patient follow-up, limiting comprehensive understanding of the full-spectrum clinical influence of preoperative carbohydrate administration.

### Regional differences: the logic of “localization adaptation” and “proximity selection” under core consensus

Preoperative oral carbohydrate intervention protocols display unified core clinical principles alongside regionally tailored implementation details, and the observable regional variations may be explained by a consistent logic of localized adaptation and proximity-based formulation selection shaped by regional medical resources, population metabolic characteristics and mainstream surgical models. Global published research converges on three shared consensus principles for carbohydrate loading interventions. Formulations dominated by maltodextrin are widely preferred given their moderate glycemic index, steady intestinal absorption rate and favorable gastrointestinal tolerance. Standard administration timing aligns with physiological gastric emptying rules, with carbohydrate intake scheduled 2–3 h preoperatively and total carbohydrate dosage controlled within the range of 25–50 g per session. In addition, all regional research maintains strict safety prerequisites confirming no elevated aspiration risk associated with standardized carbohydrate protocols, consistent with the core requirements of international ERAS guidance systems. Despite unified foundational intervention logic, cross-regional adaptive adjustments arise from proximity-based resource selection. Formulation supply represents the primary source of localized variation: research conducted within China prioritizes domestically commercialized carbohydrate beverages, and several tertiary institutions develop self-compounded liquid formulations to stabilize supply chains and reduce medical expenditure. European and American investigations mostly utilize internationally standardized commercial nutritional preparations with complete labeling of osmotic pressure and electrolyte composition, while East Asian studies from Japan and South Korea mainly adopt local proprietary zero-fasting beverage series customized for regional patient populations. Regionally available natural carbohydrate sources are occasionally incorporated into trial schemes in tropical Southeast Asian areas, representing localized adjustments based on easily accessible regional raw materials.

Surgical practice conventions in distinct geographical areas further fine-tune dosing volumes and administration modes. Hospitals in China, where laparoscopic minimally invasive operations predominate, commonly implement single-dose regimens of 200–400 mL administered 2–3 h before surgery, matching the rapid recovery trajectory of minimally invasive procedures. European medical centers performing high volumes of open major abdominal surgery frequently adopt dual pre-operative dosing to mitigate prolonged preoperative fasting discomfort. Japanese and South Korean clinical research, centered on endoscopic minor operations, favors smaller-volume 200 mL single-dose preparations to balance intervention efficacy and clinical operational convenience. No single regional protocol demonstrates absolute universal superiority over alternatives; instead, diversified localized regimens enrich the overall evidence base of preoperative carbohydrate management and provide translatable adaptive references for clinical promotion across regions with disparate medical resource conditions and surgical landscapes. The proximity-oriented selection logic strengthens the clinical operability and scalable popularization of carbohydrate loading strategies under varied local healthcare environments.

### Research limitations and directions for innovation and development

#### The main limitations of the current study

Evidence collated from the 82 included studies reveals several observable constraints that may limit the generalizability and interpretation of mapped research trends. Apart from sparse long-term postoperative outcome data and insufficient evidence focused on complex high-risk patient subgroups, five interrelated categories of limitations are summarized, covering trial methodology, recruited study populations, geographic publication imbalance, cross-trial intervention heterogeneity, and restrictive predefined screening criteria.

First, methodological weaknesses of primary trials may hinder the extrapolation of research findings. Single-center studies occupy the vast majority of included literature (74 studies), while multicenter investigations only account for 8 records (19, 30, 32, 35, 37, 82, 90, 91), and merely 11 trials enroll more than 200 participants (32, 34, 36, 37, 42, 49, 51, 52, 56, 89, 91). Single-center research is highly dependent on the unique staffing, equipment and clinical management routines of a single medical institution, which creates context-specific trial environments difficult to replicate across tiered hospitals and surgical teams with varied clinical experience, thus weakening external validity. Besides, formal standardized risk-of-bias assessment was not performed for each included primary study. Without systematic bias grading, we cannot reliably distinguish high-quality rigorous trials from those prone to performance or detection bias, which may weaken the reliability of the integrated body of evidence.

Second, primary trials display obvious selection restrictions in enrolled populations, which may narrow the scope of evidence extrapolation. Most researchers tend to recruit ASA I–II patients free from chronic comorbidities as research subjects, and individuals with gastroesophageal reflux, delayed gastric emptying, diabetes or organ dysfunction are mostly ruled out during participant screening. Elective minimally invasive surgeries also constitute the primary research setting, whereas emergency, cardiac and neurosurgical cases with elevated perioperative risk are rarely investigated. This recruitment tendency may stem from investigators’ pursuit of stable and predictable trial data with fewer safety confounding factors. Nevertheless, such selective inclusion makes the accumulated evidence less applicable to the diverse, complicated patient cohorts commonly encountered in routine clinical practice.

Third, uneven geographic distribution of publications and marked heterogeneity in carbohydrate intervention schemes jointly restrict cross-regional comparative analysis. Observably, Asian studies dominate the evidence base, while research from Europe, America and other regions is scattered and insufficient. This imbalance may arise from inconsistent popularity of ERAS protocols and varying clinical acceptance of preoperative oral carbohydrate loading across different medical regions. Additionally, the literature search covered both Chinese and English databases, and only articles published in these two languages were eligible for inclusion; such language-based inclusion boundaries may further amplify the overrepresentation of Asian studies and limit access to clinical data published in other languages. Meanwhile, intervention parameters including carbohydrate dosage, administration volume, timing and formula composition differ widely among trials. Such inconsistent study designs may be attributable to locally available nutritional preparations and institutional fasting protocols, hindering consistent cross-study interpretation of intervention efficacy and safety profiles and limiting the establishment of generalized perioperative implementation guidance.

Fourth, existing intervention schemes lack individualized adjustment rules, and relevant mechanistic exploration remains inadequate. Most trials adopt fixed carbohydrate dosage and administration timing without adjusting regimens according to patient weight, basal metabolism, gastric emptying capacity or surgical trauma severity. This uniform intervention mode may originate from researchers’ intention to simplify trial implementation and reduce confounding variables, but it fails to reflect the precision, patient-centered core concept of modern ERAS. In addition, current research priorities lean heavily toward short-term perioperative clinical indicators, with limited in-depth exploration of metabolic regulatory and anti-inflammatory mechanisms of preoperative carbohydrate intake. This predominant focus on short-term outcomes can be explained by multiple inherent practical and methodological constraints. First, preoperative oral carbohydrate loading is essentially a short-term perioperative optimized intervention targeting immediate surgical stress and early postoperative recovery, which inherently restricts its research focus to proximal perioperative endpoints. Second, long-term outcome monitoring requires prolonged patient follow-up, which imposes higher time cost, economic expenditure and patient loss-to-follow-up risks, making large-scale long-term observational trials less feasible in perioperative research. Third, preoperative carbohydrate loading is always implemented as one component of multimodal ERAS protocols rather than an independent therapeutic measure, rendering it methodologically challenging to isolate its exclusive long-term clinical benefits and causal relationships with delayed postoperative complications or long-term functional recovery. The lack of mechanistic evidence makes it hard to explain the intrinsic correlation between carbohydrate loading and clinical recovery, and insufficient long-term follow-up data also prevents comprehensive evaluation of sustained postoperative rehabilitation and late complication risks.

Fifth, predefined strict exclusion criteria inevitably restrict the exploratory breadth of this scoping review, which merits explicit acknowledgment. First, studies enrolling fewer than 20 participants were excluded to avoid unstable, underpowered data derived from case series and preliminary pilot trials. However, this threshold deviates from conventional scoping review methodology and discards small-scale exploratory trials that may record unique preliminary safety observations for preoperative carbohydrate interventions. Second, trials focusing solely on patients with decompensated diabetes, morbid obesity and advanced liver dysfunction were excluded given inconsistent perioperative safety monitoring strategies in such cohorts. As a result, the current evidence map lacks sufficient data regarding carbohydrate loading protocols for these high-risk multimorbid populations, limiting our ability to characterize intervention patterns applicable to complex clinical subgroups. Third, studies with disclosed industry-related financial conflicts of interest were excluded to reduce the risk of selective outcome reporting bias (97). This filtering step slightly narrows the scope of included evidence, though only one relevant article was removed in the full-text screening phase, exerting a minimal overall impact on the integrity of our mapped research landscape. Collectively, these rigorous predefined filters narrow the overall coverage of published literature and may obscure preliminary, underdeveloped research directions worthy of subsequent systematic exploration.

Despite such deficiencies in current primary research, this review still systematically maps the overall distribution and developmental trends of preoperative oral carbohydrate administration protocols.

### Innovative directions and future perspectives for research

Consistent with the five categories of methodological and evidence constraints elaborated in the Limitations section, the future research agenda is organized into two interconnected parts. The first part outlines actionable research priorities directly generated from gaps observed in the 82 mapped studies, requiring minimal speculative inference. The second part summarizes emerging innovative research avenues supported by limited preliminary published data; these directions remain understudied in the current body of evidence and require further validation through dedicated trials.

#### Actionable research priorities derived from mapped evidence limitations

This subsection proposes targeted, evidence-based optimization schemes for future clinical trial design, directly responding to the core evidence gaps summarized in the Limitations section.

First, corresponding to the methodological defects of single-center dominance and insufficient quality evaluation in existing studies, future trials should enhance methodological rigor through large-scale multicenter enrollment and standardized risk-of-bias assessment. Investigators ought to conduct prospective multi-institutional trials covering medical facilities at different levels to improve cross-setting generalizability. Uniform bias assessment frameworks should be embedded into trial protocols to further improve the robustness and credibility of cumulative evidence regarding preoperative carbohydrate loading.

Second, to address the prominent selection bias toward low-risk surgical populations identified in current evidence, future trial recruitment frameworks should be appropriately expanded. Specialized subgroup trials targeting underrepresented ASA III cohorts, including patients with decompensated diabetes, advanced liver dysfunction, and morbid obesity, are urgently needed. Such trials should adopt unified perioperative monitoring protocols, including standardized gastric emptying ultrasonography and dynamic glycemic assessment, to fill the existing evidence gap for high-risk multimorbid surgical patients.

Third, in response to the unbalanced regional distribution and substantial inter-protocol heterogeneity observed across mapped studies, cross-regional collaborative research should be prioritized. Future multinational multicenter studies may establish a unified core intervention standard, consisting of 25–50 g maltodextrin-based carbohydrate solutions administered 2–3 h preoperatively, while allowing flexible formulation adjustments according to local nutritional supply conditions. This hybrid framework will effectively reduce regional evidence fragmentation and facilitate the formulation of adaptable, generalized perioperative guidance.

Fourth, aiming at the lack of individualized regimens and long-term outcome data in current literature, future trial designs should integrate personalized intervention strategies and full-cycle graded follow-up protocols. Fixed, one-size-fits-all carbohydrate administration schemes should be replaced with dynamically adjusted regimens based on individual body characteristics, gastrointestinal function, and surgical trauma severity. Furthermore, multi-stage follow-up covering short-term (1 month), medium-term (3–6 months), and long-term (1–3 years) postoperative timepoints is recommended to capture sustained metabolic changes, postoperative cognitive function, and long-term oncological prognosis, providing comprehensive clinical evidence for future mechanistic exploration and protocol optimization.

#### Emerging frontier research avenues supported by preliminary literature

Beyond the above improvements targeting existing evidence gaps, several innovative and understudied research directions with preliminary preclinical and pilot data warrant further exploration to advance the refinement and translational development of preoperative carbohydrate intervention.

##### Combined intervention of preoperative carbohydrate loading and intestinal microbiota regulation

Future clinical trials can explore synergistic regimens integrating preoperative carbohydrate supplementation with intestinal microecological modulation. Such studies should focus on verifying whether combined nutritional and probiotic/prebiotic interventions regulate short-chain fatty acid metabolism, alleviate postoperative insulin resistance, and optimize surgical stress response, particularly for obese and diabetic patients with preoperative intestinal dysbiosis. This innovative combination strategy can further refine subgroup-specific perioperative nutritional management protocols (98, 99).

##### Development and clinical verification of specialized low-glycemic sustained-release carbohydrate formulations

Future research should prioritize the clinical validation of novel low-glycemic and sustained-release carbohydrate formulas. Structured controlled trials are recommended to compare the glycemic stability, safety, and postoperative recovery benefits of optimized slow-release preparations vs. conventional maltodextrin solutions. Such evidence will support the establishment of stratified, population-specific carbohydrate formulations for high-risk hyperglycemic and obese surgical cohorts (3, 13, 72).

##### Interaction analysis between preoperative carbohydrate loading and perioperative anesthetic regimens

Future perioperative research can expand multimodal exploration by focusing on the interactive relationship between carbohydrate loading and anesthetic management. Clinical studies are encouraged to clarify whether standardized preoperative carbohydrate regimens can mitigate anesthesia-induced hyperglycemia and inflammatory responses under different anesthetic modalities. The findings will facilitate the construction of individualized combined nutrition-anesthesia perioperative optimization strategies (61, 100).

##### Multi-omics mechanistic exploration and machine learning predictive modeling

Future translational research should incorporate multi-omics technologies and artificial intelligence tools to promote mechanistic and intelligent upgrading of carbohydrate intervention. Multi-omics profiling is recommended to systematically characterize glycolytic and inflammatory signaling pathways, establishing a complete “formula-mechanism-population” regulatory framework. Large-scale prospective clinical datasets should also be collected to train and validate machine learning models, achieving accurate, individualized, and intelligent preoperative carbohydrate regimen formulation in clinical practice (97, 101, 102).

### Clinical practice optimization recommendations

Medical institutions can adopt a balanced implementation principle combining universal consensus standards and localized adjustment. The core unified protocol summarized from included studies recommends maltodextrin-based carbohydrate drinks with a total carbohydrate load of 25–50 g, administered 2–3 h before surgery. For high-risk multimorbid patients lacking unified trial evidence, a closed-loop perioperative assessment workflow is suggested, including preoperative glycemic and gastric emptying screening, dynamically adjusted carbohydrate regimens, intraoperative metabolic monitoring, and postoperative follow-up. Hospitals can formulate institutional standard operating procedures matching their dominant surgical types and local patient demographics. Targeted nursing training focusing on gastrointestinal ultrasound assessment and individualized nutritional adjustment is also essential to translate ERAS evidence into routine ward practice.

## Conclusion

This scoping review systematically included 82 eligible studies published between 2015 and 2025, aiming to map the global research landscape, implementation characteristics and evidence distribution of preoperative oral carbohydrate loading within ERAS protocols.

Collectively, the included studies revealed unified standardized specifications for routine preoperative carbohydrate intervention, with maltodextrin-based formulations being the most widely adopted regimen at a proportion of 37.8%. The standard intervention consisted of 25–50 g carbohydrates dissolved in 200–400 mL liquid, administered 2–3 h preoperatively. All included clinical observations reported acceptable gastric residual volume below the safety threshold without elevated aspiration risks. In terms of evidence distribution, publications were geographically dominated by Asian studies, and research subjects mainly focused on low-risk elective adult surgical patients, while literature targeting patients complicated with diabetes, obesity or organ dysfunction remained scarce. Temporal shifts in research focus were also observed: early studies before 2020 centered on general efficacy verification, whereas post-2020 research trended toward refined safety evaluation and individualized perioperative management. Uniform core administration standards have been adopted worldwide, with minor variations in preparations and dosage strategies across medical regions.

In brief, the current body of evidence establishes a standardized core framework for preoperative oral carbohydrate loading. Obvious heterogeneity exists across study populations, intervention protocols and outcome indicators, which identifies notable research gaps to guide further clinical exploration.

## Data Availability

The original contributions presented in this study are included in this article/supplementary material, further inquiries can be directed to the corresponding author.
